# Gpr176 is a Gz-linked orphan G-protein-coupled receptor that sets the pace of circadian behaviour

**DOI:** 10.1038/ncomms10583

**Published:** 2016-02-17

**Authors:** Masao Doi, Iori Murai, Sumihiro Kunisue, Genzui Setsu, Naohiro Uchio, Rina Tanaka, Sakurako Kobayashi, Hiroyuki Shimatani, Hida Hayashi, Hsu-Wen Chao, Yuuki Nakagawa, Yukari Takahashi, Yunhong Hotta, Jun-ichirou Yasunaga, Masao Matsuoka, Michael H. Hastings, Hiroshi Kiyonari, Hitoshi Okamura

**Affiliations:** 1 Department of Systems Biology, Graduate School of Pharmaceutical Sciences, Kyoto University, Sakyo-ku, Kyoto 606-8501, Japan; 2 Core Research for Evolutional Science and Technology, Japan Science and Technology Agency, Saitama 332-0012, Japan; 3 Laboratory of Virus Control, Institute for Virus Research, Kyoto University, Sakyo-ku, Kyoto 606-8507, Japan; 4 Division of Neurobiology, Medical Research Council Laboratory of Molecular Biology, Cambridge CB2 0QH, UK; 5 Animal Resource Development Unit and Genetic Engineering Team, RIKEN Center for Life Science Technologies, Kobe 650-0047, Japan

## Abstract

G-protein-coupled receptors (GPCRs) participate in a broad range of physiological functions. A priority for fundamental and clinical research, therefore, is to decipher the function of over 140 remaining orphan GPCRs. The suprachiasmatic nucleus (SCN), the brain's circadian pacemaker, governs daily rhythms in behaviour and physiology. Here we launch the SCN orphan GPCR project to (i) search for murine orphan GPCRs with enriched expression in the SCN, (ii) generate mutant animals deficient in candidate GPCRs, and (iii) analyse the impact on circadian rhythms. We thereby identify Gpr176 as an SCN-enriched orphan GPCR that sets the pace of circadian behaviour. Gpr176 is expressed in a circadian manner by SCN neurons, and molecular characterization reveals that it represses cAMP signalling in an agonist-independent manner. Gpr176 acts independently of, and in parallel to, the Vipr2 GPCR, not through the canonical Gi, but via the unique G-protein subclass Gz.

## Abstract (web summary)

The suprachiasmatic nucleus (SCN) is the central regulator of circadian rhythms. Here the authors identify mouse Gpr176 as a pace modulator of this circadian clock and characterize its mode of action as coupling to Gz rather than Gi subunits.

## Graphical abstract

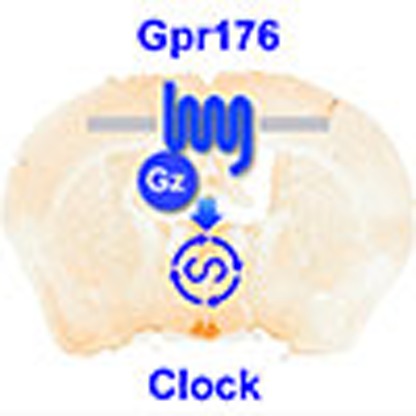

In mammals, the principal circadian pacemaker governing daily rhythms in behaviour and physiology resides in the suprachiasmatic nucleus (SCN) of the hypothalamus^1^,^2^. Most tissues outside the SCN also contain local clocks (the so-called peripheral clocks), and their rhythms are synchronized, harmoniously, by an array of direct or indirect signals from the SCN^2^,^3^. Thus, the SCN lies at the top of a hierarchical, multioscillator system distributed across the body^4^,^5^.

Malfunction of the circadian clock has been linked to the pathogenesis of a wide variety of diseases^6^, including sleep-wake disorder, tumorigenesis, obesity, diabetes and hypertension. Drug efficacy and toxicity are also under circadian regulation^7^. These lines of evidence support the potential value of developing drugs that target the circadian clock, and pioneer studies have already identified synthetic compounds that selectively target the key intracellular clock components, cryptochromes (Cry1 and Cry2)^8^ and REV-ERBα and β^9^. Because their targets are distributed across the body, these compounds can modulate both the central and peripheral clocks equally^8^,^9^. In contrast, the development of drugs that specifically target the SCN remains an unfulfilled opportunity for circadian pharmacology^4^,^7^.

G-protein-coupled receptors (GPCRs) constitute the largest family of cell surface receptors, participating in a broad range of physiological functions. It has been appreciated that GPCRs are the most common target of pharmaceutical drugs: more than 30% of clinically marketed drugs target GPCR function^10^. Intriguingly, there are still >140 orphan GPCRs whose cognate ligands are not known, and deciphering their physiological function remains a priority for both clinical and fundamental research^11^,^12^,^13^,^14^,^15^. We speculated that some orphan GPCRs, whose physiological functions have remained unknown, might exist in the SCN and function as potential modulators of the circadian system.

Structurally, GPCRs possess two different conformations, active and inactive. Agonists lock the receptor structure in its active form, antagonists block agonist action, and inverse agonists stabilize the receptor in its inactive form. In the absence of ligands, GPCRs spontaneously interchange between the two conformations; active and inactive, generating agonist-independent baseline activity^16^,^17^,^18^. Although the magnitude of this spontaneous activity differs strikingly between GPCRs, some of the orphan GPCRs exhibit significant levels of intrinsic activity^19^,^20^.

Depending on the type of G-protein to which the GPCR is coupled, a variety of downstream signalling pathways can be activated. Circadian fluctuation of cAMP signal is crucial for the maintenance of circadian clock function in the SCN^21^. In this context, the Vipr2 GPCR for vasoactive intestinal peptide (Vip) is a positive regulator of cAMP^22^,^23^ and demonstrated to be necessary for SCN time-keeping^24^,^25^. Yet, much less is known about the molecular identity of GPCR that negatively regulates cAMP production in the SCN.

cAMP synthesis is positively or negatively regulated by Gs or Gi family members, respectively. While the Gs family contains two subtypes (Gs1 and Gs2), the inhibitory members include three Gi (Gi1, Gi2, and Gi3) and one Gz. All Gi members, except Gz, are substrates of pertussis toxin (PTX). PTX mediates ADP ribosylation at the carboxyl terminal cysteine residue (−4 position), inhibiting Gi activity. Because Gz lacks this cysteine residue, it can inhibit adenylyl cyclase activity in a PTX-insensitive manner^26^. Differing from Gi, Gz is expressed mainly in the brain^27^,^28^, but its roles in the brain are not understood.

In the present study we surveyed all known orphan GPCRs expressed in the SCN, and identified three SCN-enriched genes: *Gpr176*, *Gpr19* and *Calcr*. We generated knockout mice for each of them and demonstrated that Gpr176 is a unique orphan GPCR that can set the pace of circadian behaviour. Gpr176 is expressed mainly in the brain, with prominent expression in the SCN, and its protein abundance fluctuates in a circadian fashion. Molecular characterization further revealed that this orphan receptor has an agonist-independent basal activity to repress cAMP production. Notably, the unique G-protein subclass Gz, but not the canonical Gi, is required for the activity of Gpr176. We show that Gpr176 (negative regulator of cAMP) acts independently of, and additively with, the Vipr2.

## Results

### The SCN-orphan GPCR project identifies *Gpr176*

**Figure 1:**
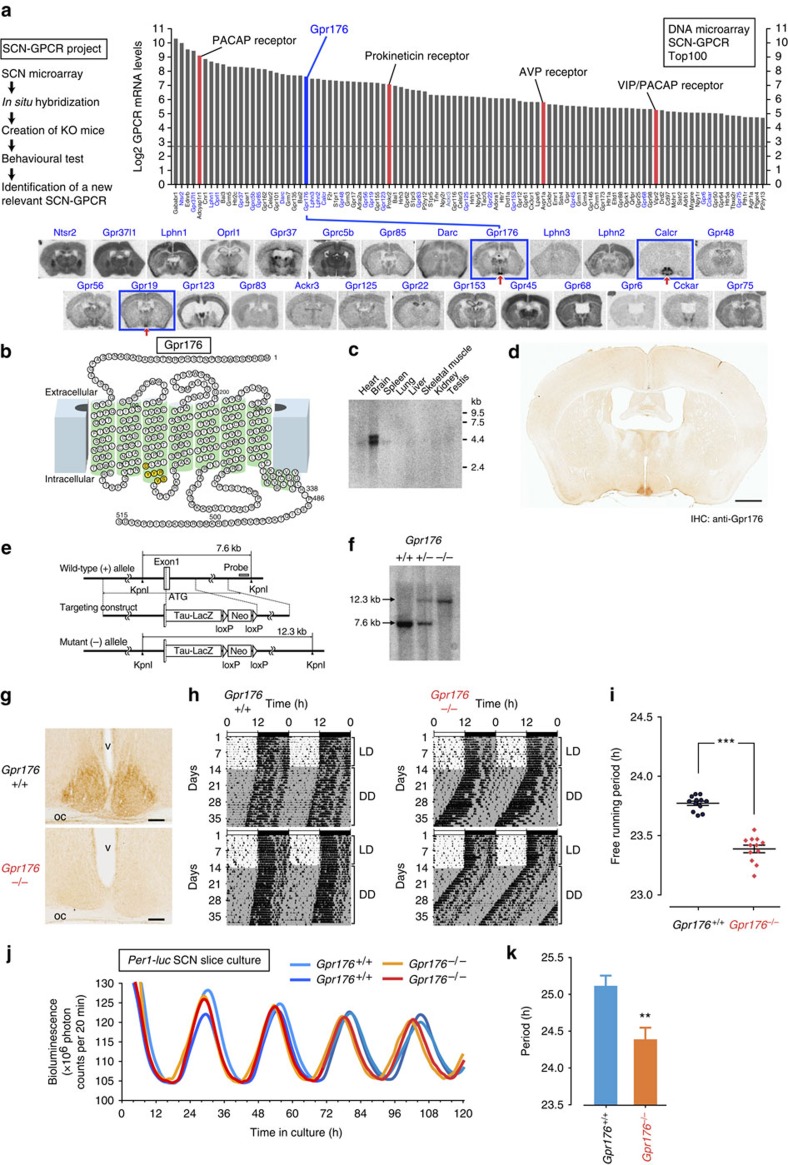
Gpr176 is an SCN-enriched orphan GPCR that sets the pace of circadian timing. (**a**) The SCN-GPCR project leading to the identification of *Gpr176*. The bar graph shows the rank order of expression of the top 100 GPCRs (classes A, B, and C) detected in the SCN microarray analysis (GEO accession number: GSE28574). Red bars are the receptors known to be expressed in the SCN. The horizontal line indicates the threshold of statistical significance of expression. The genes highlighted in blue, which include all listed orphan GPCRs, were characterized further by *in situ* hybridization using radiolabeled gene-specific probes. Arrows indicate robust positive SCN signals for *Gpr176*, *Calcr* and *Gpr19*. (**b**) Snake-plot representation of the mouse Gpr176. The residues highlighted in yellow indicate the DRYxxV motif located at the cytoplasmic end of the transmembrane helix III. (**c**) Northern blotting for *Gpr176* with a mouse multiple-tissue blot (Clontech). (**d**) Representative mouse coronal brain section immunolabeled for Gpr176. Scale bar, 1 mm. (**e**) Schematic representations of the mouse *Gpr176* gene, targeting construct, and the resulting mutant allele. A genomic region downstream of the start codon (ATG) of *Gpr176* was deleted. Grey box: probe used for Southern blot. (**f**) Southern blot of *Kpn*I-digested DNA from *Gpr176*^+/+^, *Gpr176*^+/−^ and *Gpr176*^−/−^ mice. Genomic fragments from the wild-type (7.8 kb) and mutant (12.3 kb) alleles are indicated. (**g**) Immunohistochemical confirmation of Gpr176 deficiency in the SCN of *Gpr176*^−/−^ mice. Scale bar, 100 μm. oc, optic chiasm; v, third ventricle. (**h**) Representative locomotor activity records of C57BL/6J-backcrossed *Gpr176*^+/+^ and *Gpr176*^−/−^ mice. Mice were housed in LD and then transferred to DD. Periods of darkness are indicated by grey backgrounds. Data are shown in double-plotted format. Each horizontal line represents 48 h; the second 24-h period is plotted to the right and below the first. (**i**) Circadian periods of free-running activities in DD. Periods of individual mice are plotted. Bars indicate mean±s.e.m. (*n*=12, for each genotype). ***P<0.001, Student's *t*-test. (**j**) Representative *Per1*-*luc* bioluminescence records from organotypic SCN slices of *Gpr176*^+/+^ (light and dark blue traces) and *Gpr176*^−/−^ (red and orange traces) mice. (**k**) Periods of *Per1*-*luc* rhythm in *Gpr176*^+/+^ and *Gpr176*^−/−^ SCN slices (means±s.e.m., *n*=4 for each). **P<0.01, Student's *t*-test.

We first constructed a list of GPCR genes that are potentially enriched in the SCN (Fig. 1a). Using SCN microarray data^29^ we surveyed all known mouse non-odorant GPCR genes and rank-ordered them based on their relative levels. The list of the top 100 genes (Fig. 1a) identified 23 orphan GPCRs, whose *in vivo* functions remain unknown. The receptors with known key functions in the SCN are also included in this list, for example, Adcyap1r1 (refs 30, 31), Prokr2 (refs 32, 33), Avpr1a (refs 34, 35) and Vipr2 (refs 24, 25), suggesting the relevance of the screen.

The genes highlighted in blue in Fig. 1a were further analysed by *in situ* hybridization. These include all listed orphan GPCRs (*Gpr37l1*, *Lphn1*, *Gpr37*, *Gprc5b*, *Gpr85*, *Darc*, *Gpr176*, *Lphn3*, *Lphn2*, *Gpr48*, *Gpr56*, *Gpr19*, *Gpr123*, *Gpr83*, *Ackr3*, *Gpr125*, *Gpr22*, *Gpr153*, *Gpr45*, *Gpr68*, *Gpr6*, *Cckar* and *Gpr75*) and three selected GPCRs whose roles in the SCN are not known (*Ntsr2*, *Oprl1*, and *Calcr*). To specify their histological distribution, we labelled mouse brain sections with gene-specific probes, and relatively strong, SCN-specific signals were observed for *Gpr176*, *Gpr19* and *Calcr* (Fig. 1a). On the other hand, the remaining genes were either only faintly expressed in the SCN or located more broadly in the regions outside of the SCN. Thus, based on this intensity and regionality, we selected *Gpr176*, *Gpr19* and *Calcr* as candidates of interest.

We generated knockout mice for *Gpr176*, *Gpr19* and *Calcr* (Fig. 1e,f, see also Methods). Homozygous deletion of each of them did not cause any gross abnormalities, lethality or infertility, allowing for circadian locomotor activity tests on respective adult mutant mice. Animals were entrained on a 12-h light:12-h dark (LD) cycle for 2 weeks and then transferred to constant darkness (DD) to monitor the endogenous SCN-driven locomotor activity rhythm. We found that whereas *Gpr176*-deficient mice exhibited a significantly shorter circadian period compared with wild-type littermate mice, mice lacking *Gpr19* or *Calcr* did not show any significant difference in period by genotype (circadian periods (h), mean±s.e.m. (*n*=6 for each genotype); for *Gpr176*^+/+^, 23.78±0.04; *Gpr176*^−/−^, 23.36±0.04 (*P*<0.01); for *Gpr19*^+/+^, 23.79±0.04; *Gpr19*^−/−^, 23.92±0.07 (*P*>0.1); for *Calcr*^+/+^, 23.84±0.03; *Calcr*^−/−^, 23.86±0.04 (*P*>0.1); Student's *t*-test). Importantly, *Gpr176* mutant mice that had been backcrossed to the C57BL/6J background over 10 generations also had a similarly short-period phenotype (Fig. 1h; period (*χ*^2^ periodogram)±s.e.m: *Gpr176*^−/−^, 23.39±0.03 h; *Gpr176*^+/+^, 23.77±0.02 h (*n*=12, both genotypes); Fig. 1i, *P*<0.001, Student's *t*-test). On the other hand, as shown in Supplementary Fig. 1, *Gpr176*-deficient mice were normal for light-induced phase resetting; their resetting response to early- and late-night light pulses were indistinguishable from wild-type mice. Thus, *Gpr176* is not likely to be a light signal-related receptor for the SCN. Rather, this orphan receptor appears to be involved in the determination of intrinsic period of the SCN.

To complement the data on circadian behaviour, we performed real-time bioluminescence imaging of cultured SCN slices from wild-type and *Gpr176*^−/−^ mice carrying a *Per1*-promoter-luciferase (*Per1-luc*) reporter gene^36^ (Fig. 1j,k). Crucially, bioluminescence rhythm of the *Gpr176*^−/−^ SCN slice had a shorter period than that of the wild-type control (*Gpr176*^+/+^=25.11±0.14 h; *Gpr176*^−/−^=24.39±0.16 h; mean±s.e.m., *n*=4 for each, *P*<0.01, *t*-test); the short-period phenotype of behaviour is thus ascribable to the SCN. In contrast, the short period was not observed for their lung slices (*Gpr176*^+/+^=24.76±0.09 h; *Gpr176*^−/−^=24.81±0.31 h; *n*=4 for each), suggesting that the effect of *Gpr176* deficiency may be specific to the central oscillator.

Of note, northern blot analysis with different tissues (Fig. 1c; Supplementary Fig. 2a,b) revealed that *Gpr176* messenger RNA (mRNA) is expressed chiefly in the brain. *In situ* hybridization was therefore further conducted to map the neural expression from the forebrain to the medulla oblongata (Supplementary Fig. 2c). Notably, the highest expression of *Gpr176* was observed within the SCN. Relatively strong and restricted expression was also seen in the subfornical organ (SFO), organum vasculosum of the lamina terminalis (OVLT) and cerebellar flocculus (Fl), among other regions, suggesting that *Gpr176* in extra-SCN sites may contribute to different brain functions that we have not yet systematically investigated.

### Clock-controlled circadian expression of *Gpr176* in the SCN

*Gpr176* encodes a class A orphan GPCR^37^(Fig. 1b), whose *in vivo* function is unexplored. To further investigate its role in the circadian system, we profiled temporal expression of *Gpr176* in the SCN (Supplementary Fig. 2d). To do this, we performed quantitative *in situ* hybridization, using mice housed either in LD or DD, their brains being collected at 4-h intervals across 24-h cycles. Notably, in both LD and DD, the SCN had a time-of-day-specific expression of *Gpr176* (Supplementary Fig. 2d). In LD, *Gpr176* was highest at night at ZT16 (ZT represents *Zeitgeber* time; ZT0 denotes lights-on and ZT12 lights-off) and lowest in the early morning at ZT0 (*P*<0.01, peak versus trough, one-way analysis of variance (ANOVA) with Bonferroni *post hoc* test). Similarly, in DD, *Gpr176* was highest in the subjective night at CT16 (CT represents circadian time; CT0 denotes the beginning of the subjective day and CT12 the beginning of the subjective night) and lowest in the subjective morning at CT4 (*P*<0.01, peak versus trough). Furthermore, we found that the subjective-night peak expression of *Gpr176* was severely damped in mice deficient in the core clock components *Cry1* and *Cry2*, which completely lack a functional circadian clock^38^,^39^ (Supplementary Fig. 2e), indicating that the SCN clockwork controls *Gpr176* gene expression. A light pulse given at night did not cause any acute change in *Gpr176* expression in the SCN (Supplementary Fig. 2f). Thus the SCN appears to direct *Gpr176* expression independently of light.

### Characteristic distribution of Gpr176 across the whole SCN

We developed an affinity-purified rabbit polyclonal antibody against Gpr176 and performed immunohistochemistry. Coronal brain sections from wild-type mice (Fig. 1d) revealed marked SCN-specific immunoreactivity to Gpr176 (see also Supplementary Fig. 3a), while little or no immunostaining was observed for *Gpr176*^−/−^ SCN (Fig. 1g; Supplementary Fig. 3a).

The SCN is composed of anatomically heterogeneous subregions^1^,^40^. We thus studied the topographical distribution of Gpr176 by staining serial coronal brain sections covering the whole rostral-caudal extent of the SCN (Supplementary Fig. 3c). Positive immunostaining of Gpr176 was widely observed from the rostral to caudal extremities of the SCN. Moreover, we noticed that the immunoreactivity to Gpr176 was relatively strong in the dorsomedial area of the SCN, a region also referred to as the SCN ‘shell'^41^. This characteristic distribution profile was reminiscent of the Vipr2 receptor for Vip^42^. Interestingly, anti-Vipr2 immunoreactivity in the SCN, revealed by rabbit polyclonal antiserum, was widespread, extending from the rostral to caudal margins, and more intense in the dorsomedial than the ventrolateral region of the SCN (Supplementary Fig. 3b,c)^42^.

Immunolocalization of Gpr176 was also compared with that of neuropeptide markers in the SCN. We performed double-label confocal immunohistochemistry (Supplementary Fig. 4) and found that the two non-overlapping SCN populations, Vip- and vasopressin (AVP)-ergic neurons, both appear to express Gpr176. Interestingly, Vipr2 is also expressed in both populations^42^.

### Histological relationship between Gpr176 and Vipr2

The resemblance of immunohistological distribution of Gpr176 to that of Vipr2 attracted our attention. Vipr2 is a class C GPCR that serves as a Vip receptor in the SCN and has already been shown to play a key role in circadian pacemaking^24^,^25^. We thus clarified whether Gpr176 colocalizes with this important receptor.

**Figure 2:**
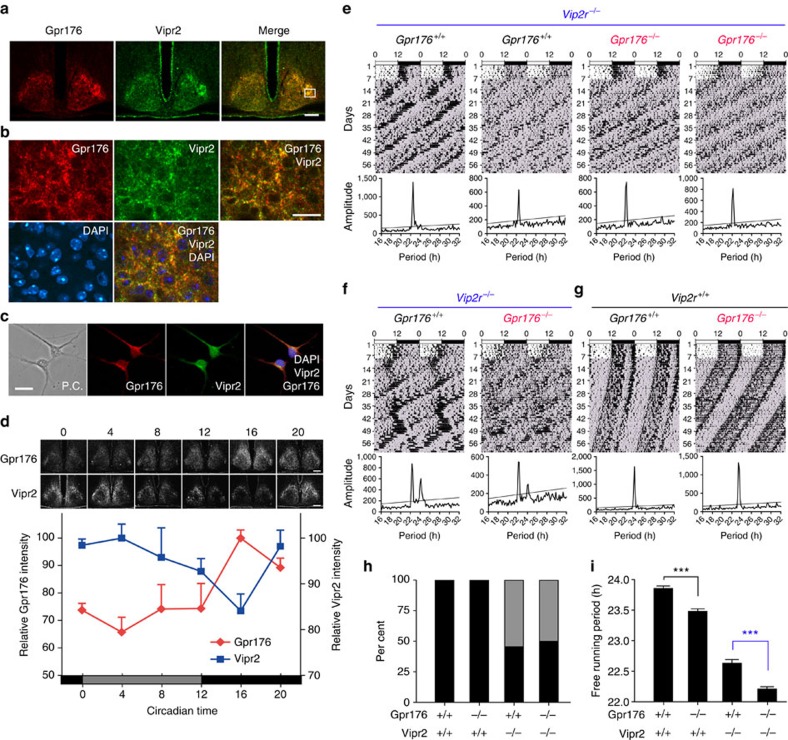
Characterization of histological and genetic relationships between Gpr176 and Vipr2. (**a**) Double-label immunofluorescence of Gpr176 and Vipr2 in the mouse SCN. Coronal brain sections were immunolabeled with antibodies against Gpr176 (rabbit polyclonal) and Vipr2 (chicken polyclonal). Representative confocal pictures are shown, with a merged image of Gpr176 (red) and Vipr2 (green). Scale bar, 100 μm. (**b**) Enlargement of the boxed area in (**a**). Merge shows combined images for Gpr176 (red), Vipr2 (green), and DAPI-based nuclear staining (blue). Scale bar, 20 μm. (**c**) Immunofluorescence and phase contrast (P.C.) images of dispersed SCN neurons. Cells were immunolabelled for Gpr176 and Vipr2. Merge shows combined images of Gpr176 (red), Vipr2 (green), and DAPI (blue). Scale bar, 20 μm. (**d**) Antiphasic circadian expression profiles of Gpr176 and Vipr2 in the SCN. Values (mean±s.e.m.) indicate relative immunoreactivities of Gpr176 and Vipr2 at 6 time points in DD (*n*=6 brains for each data point). Representative images of the immunolabeled SCN sections are shown on the top. Scale bar, 200 μm. (**e**–**g**) Representative double-plotted actograms and *χ*^2^ periodograms of locomotor activity rhythms of mice carrying wild-type, *Gpr176*^−/−^ and *Vipr2*^−/−^ alleles. *Vipr2*^−/−^ mice exhibit a single (**e**) or multiple (**f**) circadian periods in DD, while all wild-type and *Gpr176*^−/−^ mice had a single, stable circadian period (**g**). Diagonal line on periodogram shows significance at *P*<0.001. See also Supplementary Fig. 6. (**h**) Percentage of mice expressing a single dominant circadian period (black) or multiple circadian periods (grey). *n*=8−12 for each genotype. (**i**) Group data showing individual and combined effects of *Gpr176*^−/−^ and *Vipr2*^−/−^ on circadian period of locomotor activity rhythms. Values (mean±s.e.m.) indicate free-running periods of mice with a single dominant period in DD (*n*=5−8). ***P<0.001, one-way ANOVA with Bonferroni *post hoc* test.

To visualize Vipr2 expression in the SCN, we generated anti-Vipr2 chicken polyclonal antibody, for which we confirmed specific SCN staining and its absence in mice lacking *Vipr2* (Supplementary Fig. 5). Dual-label immunofluorescence microscopy (Fig. 2a) revealed markedly overlapping immunoreactivities for Gpr176 (*red*) and Vipr2 (*green*). Both signals tended to be more intense in the dorsomedial than ventrolateral area of the SCN (Fig. 2a). Moreover, high-magnification images showed that both receptors were expressed in almost all individual cells with comparable subcellular locations (Fig. 2b). Dual-labelled cells were also observed when the SCN neurons were dispersed in culture (Fig. 2c), confirming the colocalization of Gpr176 and Vipr2.

Circadian expression profiles of Gpr176 and Vipr2 in the SCN were then compared. Brains were collected from mice at six time points in DD. Because of widespread distribution of Gpr176 and Vipr2, expression levels of each receptor were evaluated as a sum of the whole immunoreactivity from the rostral to caudal extremities of the SCN (10 sections per brain; *n*=6 mice for each data point). The results revealed that Gpr176 immunoreactivity was highest in the subjective night at CT16 and lowest in the subjective day at CT4 (*P*<0.01, peak versus trough, one-way ANOVA with Bonferroni *post hoc* test; Fig. 2d). This protein cycle is almost in phase with its mRNA expression that peaks at CT16 (Supplementary Fig. 2d,e). In comparison, Vipr2 immunoreactivity displayed an opposite circadian cycle, characterized by a robust decrease in the subjective night at CT16 (Fig. 2d) as reported previously^42^. These results illustrate that circadian profiles of Gpr176 and Vipr2 in the SCN are antiphasic.

### Genetic relationship between Gpr176 and Vipr2

We next sought to understand the relationship between Gpr176 and Vipr2 (Fig. 2e–i). To do this, we generated double deficient mice (*Gpr176*^−/−^; *Vipr2*^−/−^). By crossing double heterozygous mice (*Gpr176*^+/−^;*Vipr2*^+/−^), we obtained genetic background (C57BL/6J)-matched male wild-type (*n*=8), respective single (*n*=8 for *Gpr176*^−/−^, *n*=11 for *Vipr2*^−/−^), and double knockout (*n*=12) mice, and compared their behavioural rhythms in DD. As observed above, all single *Gpr176* knockout mice (*Gpr176*^−/−^; *Vipr2*^+/+^) had shorter periods than those of control mice (*Gpr176*^+/+^;*Vipr2*^+/+^) (Fig. 2i; Supplementary Fig. 6a,b). *Vipr2*^−/−^ mice had altered circadian rhythms as documented previously^25^,^43^ (Fig. 2e–g), displaying either greatly reduced or multiple circadian periods in DD. About half of *Vipr2*^−/−^ mice (6 of 11) had a single, short-circadian period (Fig. 2e), whereas the other half (5 of 11) simultaneously expressed two or more statistically significant circadian periods (Fig. 2f; Actograms of all individual mice are available in Supplementary Fig. 6). Concomitant deletion of *Gpr176* and *Vipr2* (*Gpr176*^−/−^;*Vipr2*^−/−^) did not change this mixed phenotype (Fig. 2e,f): about half of doubly deficient mice (6 of 12) still had a single short-circadian period (Fig. 2h), indicating that *Gpr176* is not involved in the penetrance of this phenotype. Under these conditions, we compared period length between genotypes (Fig. 2i) and found that the deletion of *Gpr176* could induce further significant shortening of the circadian period length in *Vipr2*^−/−^ background: the circadian period of double knockout mice (*Gpr176*^−/−^;*Vipr2*^−/−^) in DD was 22.20±0.03 h (*χ*^2^ periodogram, mean±s.e.m.), which was significantly shorter than that of *Gpr176* wild-type, *Vipr2* knockout mice at 22.63±0.05 h (*P*<0.001, one-way ANOVA with Bonferroni *post hoc* test). Thus, the shortening effect of *Gpr176*^−/−^ was not masked by, and acted additively with, the absence of *Vipr2*.

### Gpr176 basal activity antagonizes Vip–Vipr2–cAMP signalling

**Figure 3:**
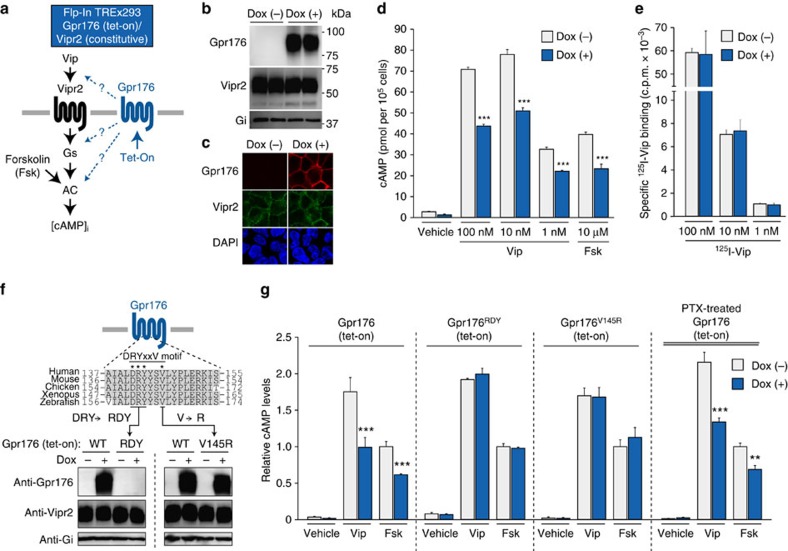
Gpr176 basal activity inhibits Vip–Vipr2–cAMP signalling. (**a**) Experimental schemes based on Flp-In TREx293-Gpr176(tet-on)/Vipr2 cells. (**b**) Immunoblots of Gpr176 and Vipr2 with cell membrane fractions of Flp-In TREx293-Gpr176(tet-on)/Vipr2 cells. Cells were treated with either 1 μg ml^−1^ Dox or control vehicle for 24 h. Gi was used as a loading control. (**c**) Confocal images of representative dual-label immunofluorescent staining of Gpr176 and Vipr2 in Dox-treated or non-treated Flp-In TREx293-Gpr176(tet-on)/Vipr2 cells. (**d**) Antagonistic basal activity of Gpr176 on Vip- and forskolin (Fsk)-stimulated cAMP accumulation in Flp-In TREx293-Gpr176(tet-on)/Vipr2 cells. Cells of the same batch were cultured in parallel with or without Dox for 24 h then resuspended in 0.5 mM IBMX (non-selective phosphodiesterase inhibitor)-containing assay buffer for 1 h and stimulated with Vip or Fsk for 15 min. Individual treatments were run in each experiment in triplicate. cAMP levels were determined by enzyme immunoassays. Values are expressed as mean±s.e.m. per 1 × 10^5^ cells. ***P<0.001 in comparison with the control stimulated group. (**e**) Unaltered, dose-dependent [^125^I]-Vip binding activities of Dox-treated Flp-In TREx293-GPR176(tet-on)/Vipr2 cells. Values are means±s.e.m. of four independent experiments. (**f**) Immunoblots of mutant cell lines expressing either Gpr176^RDY^ or Gpr176^V145R^. Point mutations were introduced into the conserved DRYxxV motif residues on Gpr176 in Flp-In TREx293-Gpr176(tet-on)/Vipr2 cells. Western blotting was done as described in **b**. (**g**) Diminished basal activities of Gpr176 by DRYxxV motif mutation. Dox-treated or non-treated cells were stimulated with Vip (100 nM), Fsk (10 μM), or vehicle then subjected to cAMP assays. Where specified, cells were treated with PTX (100 ng ml^−1^) for 16 h before assay. Each experiment was done in triplicate. cAMP values (means±s.e.m.) are plotted relative to those of Fsk-stimulated uninduced cells. ***P<0.001, **P<0.01 in comparison with the control stimulated group.

To explore potential functional interaction between Gpr176 and Vipr2 we developed a heterologous expression system (Fig. 3a). Agonist-independent constitutive function has been assigned to a number of orphan GPCRs. Thus, in the absence of a known ligand, we looked for a constitutive action or influence of Gpr176 on Vip–Vipr2-mediated signalling (Fig. 3a). We employed the Flp-In TREx293 cell system and established a clonal cell line that expressed Vipr2 constitutively (Fig. 3b,c, middle) and Gpr176 in a doxycycline-dependent, inducible manner (Fig. 3b,c, upper; see Methods for details). Confocal microscopy revealed markedly increased plasma membrane-localized immunofluorescence of Gpr176 in induced cells. On the other hand, constant membrane localization was observed for Vipr2.

Induction of Gpr176 led to an attenuation of Vip–Vipr2-mediated cAMP signalling (Fig. 3d): Vip-promoted cAMP accumulation was significantly reduced by doxycycline treatment (*P*<0.01, versus nontreatment control) at any of the dosages of Vip used for stimulation (100, 10, and 1 nM). This was not due to a difference in cell viability or cell number between doxycycline- and control-treated groups, the growth rate of the cells being equivalent in the two groups. Furthermore, we assayed the same number of cells (Fig. 3d) resuspending 3 × 10^5^ viable cells in serum-free assay buffer for stimulation with Vip.

Neither plasma membrane localization nor total protein amount of Vipr2 changed appreciably with doxycycline treatment (Fig. 3b,c). Moreover, ^125^I-labelled Vip binding was almost identical between doxycycline-treated and non-treated cells (Fig. 3e). Thus, attenuation of Vip signalling by Gpr176 induction is likely to arise from changes in the downstream pathway. In accordance with this notion, forskolin-stimulated cAMP accumulation was also blunted by the induction of Gpr176 (Fig. 3d). Forskolin is a cell-permeable drug that directly activates adenylyl cyclases. Thus, the mode of action of Gpr176 does not necessarily depend on the integrity of Vip–Vipr2. Consistent with this, Gpr176 exhibited similar basal activity when expressed alone in Flp-In TREx293 cells without concomitant expression of Vipr2 (Supplementary Fig. 7a–c).

Contrasting to the overt effect on cAMP, induction of Gpr176 did not bring about any significant change in inositol phosphate IP_1_ formation (Supplementary Fig. 7d). Thus, the downstream action of Gpr176 activity appears to be linked specifically to cAMP regulation.

Gpr176 contains a conserved Asp–Arg–Tyr–X–X–Val (DRYxxV) motif at the cytoplasmic end of transmembrane domain III (Figs 1b and 3f). Generally, this motif is important for coupling of GPCRs to the partner G-proteins. We thus wondered whether this motif is required for generation of the basal activity of Gpr176. In other GPCRs, including the related orphan receptor Gpr161 (ref. 19) as well as the other class A non-orphan GPCRs such as Cxcr1 (ref. 44) and Adra1b (ref. 45) a single point mutation of the valine residue located following the DRY triplet sequence led to a drastic loss of the receptor-mediated signalling. We therefore established a mutant Gpr176 inducible cell line where the homologous valine of this protein was mutated to arginine (V145R; Fig. 3f). Notably, regardless of the type of stimuli (Vip or forskolin), mutant Gpr176 did not yield any noticeable reduction of cAMP (Fig. 3g), although the induction of protein levels was similar to that of the wild-type (Fig. 3f), suggesting that the V145R mutation blocks Gpr176 activity. As an alternative mutation, the DRY sequence was changed to RDY (Fig. 3f)^46^, but this mutation caused a loss of detectable Gpr176 expression and as a result no associated cAMP reduction was observed (Fig. 3f,g). Thus, the DRYxxV motif seems important for both protein activity and stability of Gpr176. We generated additional mutants on the DRY triplet sequence and tested their activities using a cAMP GloSensor assay, and the results further support this conclusion (see Supplementary Fig. 8).

Pertussis toxin (PTX) has been widely used to test the involvement of Gi signalling. This toxin is able to inactivate all Gi/o family members except Gz. Importantly, PTX displayed a strong inhibitory effect on Gi-coupled sphingosine-1-phosphate (S1P) receptor signalling in TREx293 cells^47^ (Supplementary Fig. 9). However, we could not detect any noticeable effect of PTX on the Gpr176-mediated signalling (Fig. 3g; Supplementary Fig. 7c): Gpr176 still had an essentially unimpaired capacity to reduce cAMP accumulation even after PTX treatment (100 ng ml^−1^, 16 h). Thus, PTX-insensitive G-protein might mediate the downstream action of Gpr176.

### Gpr176 couples to Gz

**Figure 4:**
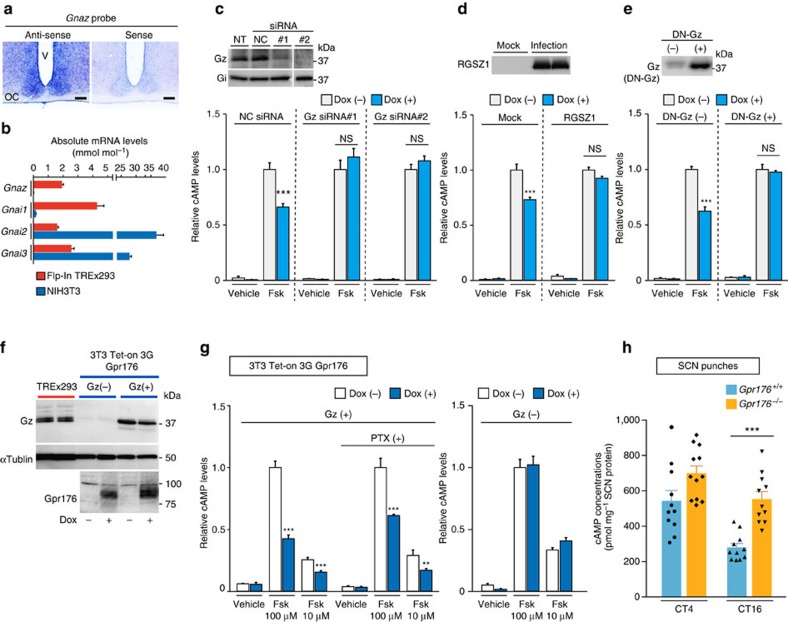
Gpr176 couples to Gz. (**a**) *In situ* hybridization of the gene encoding Gz (*Gnaz*) in the mouse SCN using a digoxigenin-labelled gene-specific probe. Scale bar, 100 μm. (**b**) Expressions of the genes encoding Gz (*Gnaz*), Gi1 (*Gnai1*), Gi2 (*Gnai2*) and Gi3 (*Gnai3*) in TREx293 (red) and NIH3T3 (blue) cells. Values (means±s.e.m.) were determined by qRT–PCR and normalized to those of the ribosomal phosphoprotein P0 (Rplp0)-encoding gene (*n*=3). (**c**) Interference of Gpr176-mediated cAMP repression by knocking down Gz protein with two different siRNA mixtures. For simplicity, Flp-In TREx293 cells bearing only Gpr176 were used (no concomitant Vipr2 expression in this assay). Cells were transfected with siRNAs for 96 h, with or without Dox for the final 24 h, and stimulated by Fsk (100 μM). Transfection was also performed with a negative-control siRNA (NC). Immunoblots show efficient knockdown of Gz. There was no off-target effect on Gi. NT, non-transfected cells. cAMP levels are plotted relative to those of Fsk-stimulated uninduced cells. Data are the mean±s.e.m. of at least two independent experiments with three replicates each. ***P<0.001 in comparison with the control stimulated group. NS, not significant. (**d**) Inactivation of Gpr176-mediated cAMP reduction by RGSZ1 coexpression. Cells were infected with lentiviruses encoding GFP (mock) or HA-tagged RGSZ1 and treated with or without Dox for 24 h. Western blot shows delivered proteins (anti-HA). cAMP values in Fsk-stimulated (100 μM) and nonstimulated (vehicle) cells are shown as described in **c**. (**e**) Interruption of Gpr176-mediated cAMP reduction by coexpression of DN-Gz. DN-Gz was stably introduced into the cells, and Dox-treated or non-treated cells were stimulated with Fsk (100 μM) or vehicle. Western blot (anti-Gz) shows the predominance of DN-Gz over the endogenous protein. cAMP values are expressed as described in **c**. (**f**) Generation and western blot confirmation of NIH3T3-based Tet-inducible Gpr176 cell lines with or without stable expression of Gz. Cells were treated with or without Dox and immunoblotted for indicated proteins. Note that Gz is scant in parental NIH3T3 cells. (**g**) cAMP repressive activities expressed by Gpr176 in NIH3T3 Gz(+) cells. Dox-treated or non-treated cells were stimulated with Fsk or vehicle. Where specified, cells were treated with PTX for 16 h before assay. cAMP values are expressed as described in **c**. (**h**) cAMP levels in the SCN of *Gpr176*^+/+^ and *Gpr176*^−/−^ mice at CT4 and CT16 in DD. SCN punches taken from mice were subjected to cAMP quantification by using a cAMP-specific enzyme immunoassay kit. Each data point represents a single animal. Bars indicate means±s.e.m. for *Gpr176*^+/+^ CT4 (*n*=11), *Gpr176*^−/−^ CT4 (*n*=12), *Gpr176*^+/+^ CT16 (*n*=11), and *Gpr176*^−/−^ CT16 (*n*=11). ***P<0.001, two-way ANOVA with Bonferroni *post hoc* test.

Gz is a unique Gi/o subfamily member that can repress adenylyl cyclases in a PTX-insensitive manner^26^, and its expression is known to be high particularly in the brain^27^,^28^ and in several specific tissues or cells in the periphery^48^. Microarray data indicate that the gene encoding Gz (*Gnaz*) is also expressed in the human embryonic kidney (HEK)293 cells^49^, a parental cell line of Flp-In TREx293 cells. We performed *in situ* hybridization and confirmed that the gene encoding Gz (*Gnaz*) is expressed in the mouse SCN (Fig. 4a; Supplementary Fig. 10). *Gnaz* was also found to be expressed in Flp-In TREx293 cells with abundance comparable to that of the other Gi family members (Fig. 4b). By contrast, the mouse embryonic fibroblast NIH3T3 cells did not express Gz (Fig. 4b,f) despite displaying high-level expression of various Gi. Akin to Gpr176, Gz is conserved among vertebrates (see http://ensembl.org/Multi/GeneTree/).

To test the hypothesis that Gz might be a mediator of Gpr176 signalling, we performed siRNA-mediated knockdown of the endogenous Gz protein in Flp-In TREx293 cells (Fig. 4c), using two different siRNA mixtures, both of which reduced the endogenous Gz protein expression levels to <10% of those observed for control siRNA treatment (NC) or nontransfection (NT) control (Fig. 4c). There was no off-target effect on the levels of Gi (Fig. 4c). Notably, both siRNAs against Gz abrogated Gpr176 activity, while negative control (NC) siRNA did not (Fig. 4c). We also used the regulator of G-protein signalling (RGS) protein family member RGSZ1, which selectively inhibits Gz^50^,^51^. We observed that lentiviral expression of this protein prevented the suppression of cAMP levels by Gpr176 (Fig. 4d). Similarly, overexpression of a dominant negative Gz (DN-Gz) protein^52^ resulted in a loss of the Gpr176-mediated cAMP reduction (Fig. 4e). Furthermore, to test this activity in a different cell system, we developed doxycycline-inducible NIH3T3 cell lines expressing Gpr176 (Fig. 4f). Because of the deficiency of Gz in NIH3T3 cells (Fig. 4b), Gz was stably introduced into the cells at levels comparable to those of Flp-In TREx293 cells (Fig. 4f, Gz(+)). Under these conditions Gpr176 displayed its effect (Fig. 4g): a significant reduction of forskolin-stimulated cAMP accumulation was observed when the cells were treated with doxycycline. In agreement with the unique property of Gz, PTX did not inhibit this reduction (Fig. 4g). Moreover, without exogenously expressed Gz, the cells did not elicit any noticeable activities of Gpr176, confirming that Gpr176 requires Gz for its activity (Fig. 4g, Gz(−)).

### Gpr176 is a negative modulator of cAMP synthesis in the SCN

The data thus far in cell culture suggest that Gpr176 is a negative regulator of cAMP signalling. Finally, we examined cAMP content in the SCN from wild-type and *Gpr176*^−/−^ mice (Fig. 4h). In agreement with the above *in vitro* data, we observed that the deletion of *Gpr176* leads to increased cAMP content in the SCN (Fig. 4h): The effect of the deletion of *Gpr176* is more evident and significant at CT16 than CT4 (*P*<0.001 for CT16, two-way ANOVA with Bonferroni *post hoc* test), while even at CT4 there is a similar trend towards increased cAMP levels in *Gpr176*^−/−^ SCN. Based on these data, we conclude that Gpr176 can act as a cAMP suppressor not only *in vitro* but also *in vivo* in the SCN.

## Discussion

In the hope of identifying a new GPCR that tunes the central clock, we searched for orphan GPCRs whose expression is enriched in the SCN. Gene knockout studies of candidate genes of interest revealed that Gpr176 is an SCN-enriched orphan GPCR required for normal circadian behaviour. Nearly all SCN neurons express Gpr176, and its abundance fluctuates in a circadian manner. Molecular characterization revealed that this orphan receptor has an agonist-independent basal activity to repress cAMP production. Notably, the unique G-protein subclass Gz, but not the canonical Gi, is required for the activity of Gpr176.

In the SCN, Gpr176 colocalizes with Vipr2. Given this overlapping expression, together with the ability of Gpr176 to compete with the Vip–Vipr2–cAMP signal, we surmise that the nighttime cAMP repression mediated by Gpr176 may serve as a part of the cAMP repressing mechanism that could counteract the Vip-Vipr2 axis in the SCN. As an additional feature of importance, Gpr176 is expressed mainly in the brain, with prominent expression in the SCN. This region specificity suggests that, as a putative target for circadian therapeutics, Gpr176 might possess the advantage of specificity, compared with broadly expressed neurotransmitter receptors, clock gene products and general modulators of second-messenger signalling such as phosphodiesterases. Thus, the results not only reveal a new signalling module, Gpr176/Gz, in the control of SCN circadian time-keeping, but also provide, thereby, a new class of GPCR signalling as a potential drug target to modulate the central clock in the brain.

In the present study, we focused on genes enriched in the SCN. The rationale for this tissue-specific approach is, first, the SCN is responsible for circadian behaviour, the ultimate and definitive arbiter of daily life^53^. Second, we considered that specificity for local mechanisms is required because the SCN differs from the peripheral clocks in phasing. In rodents, for example, the molecular clockwork of the SCN phase-leads that of peripheral clocks by 7 to 11 h (ref. 54). Any drug targeting common circadian mechanisms would be able to affect or reset all the clocks simultaneously, and this might be deleterious for keeping the adaptive phasic order between the tissues (unless such drugs were to be delivered to the target tissue selectively—a very demanding regimen). We thus reasoned that pursuing the molecular mechanisms that underpin the specificity of the SCN would be a valuable alternative way to search for potential drug targets in the central clock. Third, and last, the SCN-GENE selection strategy can be biased for ‘druggable' targets. In the current study we found Gpr176 by focusing on the GPCR family, but the project readily lends itself to other target categories, for example, ion channels, suggesting the continued relevance of this screening method, in conjunction with others, for the discovery of clock gene modulators in the SCN.

Agonist-independent basal activity has been observed for a number of ‘physiologically relevant' orphan GPCRs: for example, Gpr3, an orphan GPCR endowed with constitutive Gs signalling activity, protects oocytes from ageing^20^ and modulates amyloid-β production in neurons^55^. The orphan receptor Gpr161, which participates in Sonic Hedgehog (Shh) signalling during neural tube development, also displays constitutive activity^19^. Agonist- independent intrinsic activity is also implicated in non-orphan GPCRs' physiological function. The odorant receptors (ORs), which are GPCRs, possess an intrinsic activity and—in the absence of activating odorant—regulate axonal projection of olfactory neurons^56^. Thus, by analogous inference, intrinsic activity of Gpr176 could underlie its effect *in vivo*. Indeed, there may be no endogenous ligand, in which case the main regulatory control of activity would be via the level of protein expression, which for Gpr176 was highly circadian in the SCN. Consistent with its negative effect on cAMP, Gpr176 protein increased at night, a phase when cAMP levels in the SCN were decreased to the circadian nadir levels^21^,^29^. The absence of Gpr176, in turn, led to reduced suppression of cAMP level in the night, supporting the hypothesis. Nevertheless, the presence of unidentified endogenous ligands is always difficult to exclude for receptors with constitutive action. In this respect, it is worth noting that even without a known natural ligand, surrogate ligands can be developed for the orphan GPCRs^11^,^17^, highlighting the druggable feature of this protein family.

Previous studies demonstrated that pharmacological inhibition of cAMP synthesis in the SCN leads to longer circadian period of behavioural rhythm^21^,^29^. Compatible with this, the loss of the cAMP suppressor Gpr176 leads to the opposite phenotype, period shortening. In agreement with the reduced suppression of cAMP signal during the night, we observed that circadian rising phase of *Per1*-*luc* activity was significantly accelerated in the *Gpr176*^−/−^ SCN slices (see Supplementary Fig. 11; waveform analysis of Fig. 1j), implying that the deletion of the suppressive signal from Gpr176 allows early rising of *Per1* expression and thereby shortens circadian period. A simple explanation for the possible underlying mechanism may involve cAMP signal-mediated regulation of *Per1* transcription through a cAMP-responsive element on its promoter^57^. However, the mechanism(s) through which the circadian fluctuation of cAMP signal is integrated to the core clock machinery is still unclear in the literature and needs further exploration. In addition, a cohort of double deficient mice for *Gpr176* and *Vipr2* (*Gpr176*^−/−^; *Vipr2*^−/−^) were still rhythmic in DD, albeit with a severely shortened period of circadian locomotor activity rhythms. These data raise the possibility of potential compensatory mechanisms, perhaps through alternative GPCRs in the SCN, as previously suggested^58^. A complete understanding of circadian regulation of cAMP signalling in the SCN, and of the concerted roles of Gpr176 (repressor) and Vipr2 (activator) will be a challenge of future study.

Unlike Gi, Gz does not serve as a substrate for PTX because the consensus cysteine residue in the fourth position from the C terminus of Gi proteins is replaced with isoleucine. We showed that Gpr176 links preferentially to Gz. It is generally considered that Gz shares the same receptor coupling profile with the Gi subtypes, but there are some exceptions. For example, Smoothened (smo), which is an orphan G-protein-linked seven-transmembrane protein that mediates Hedgehog signalling, has been shown to activate Gz more drastically than Gi^59^. The mechanism of specificity remains unclear but may involve the different amino-acid sequence of the C-terminal region between Gi and Gz. Because the C-terminal region of G proteins is important for physical interactions with upstream receptors^60^, slightly higher hydrophobicity of isoleucine (Gz) over cysteine (Gi) may affect this selectivity. Finally, as a critical difference between Gz and Gi, Gz is expressed predominantly in the brain. The brain distribution of Gz is more widespread than Gpr176, implying that additional Gz-linked orphan GPCRs may remain to be identified in the brain.

Gpr176 is an evolutionally conserved, vertebrate class A orphan GPCR, initially cloned by Hata *et al*.^37^ from a human brain cDNA library. Amino-acid sequence analysis reveals that Gpr176 contains four putative glycosylation sites at the N-terminal part^37^. A relatively large, C-terminal domain of about 200 amino acids (the exact sequence is omitted in the snake plot presentation in Fig. 1b) also characterizes Gpr176. Of interest, this C-terminal cytosolic region is highly conserved among *Gpr176* genes in different species, yet does not show homology to any other annotated protein sequences, thus implying a unique feature characterizing this GPCR. Within its seven transmembrane domains, Gpr176 does not have an aspartic acid at position 2.50 (BW numbering), a feature also rarely observed for class A GPCRs. Understanding how these structural features affect molecular functions of Gpr176 will be a topic of future study.

In summary, we described here that Gpr176 is an SCN-enriched orphan GPCR that can set the pace of circadian behaviour. This is the first report to assign a function of this orphan receptor in physiology. We revealed that Gpr176 is a previously uncharacterized Gz-linked orphan GPCR that bears intrinsic activity to reduce cAMP production. The discovery of the functional orphan GPCR with a novel mode of action within the SCN would be of help to understand the mechanism that underpins the SCN and thereby facilitate searching for a potential specific drug target to modulate the central clock.

## Methods

### Mouse strains

*Gpr176*^−/−^ mice (Acc. No. CDB0672K: http://www.cdb.riken.jp/arg/mutant%20mice%20list.html) were generated in the RIKEN CDB (Kobe, Japan) and backcrossed to C57BL/6J for 10 generations. Then, *Gpr176*^+/−^ mice were intercrossed to produce homozygous null and wild-type progenies for behavioural tests. *Vipr2*^−/−^ mice^24^ were bred on C57BL/6J background^58^. A cohort of *Gpr176*^−/−^;*Vipr2*^−/−^ mice and control siblings was produced by crossing double heterozygotes (*Gpr176*^+/−^;*Vipr2*^+/−^) using *in vitro* fertilization. *Cry*-null mice (*Cry1*^−/−^;*Cry2*^−/−^) were bred as described previously^38^,^39^,^61^. Targeted mutant mice for *Gpr19* (*Gpr19*^*tm1Dgen*^) and *Calcr* (*Calcr*^*tm1Dgen*^) were obtained from the Mutant Mouse Regional Resource Center at the University of North Carolina with a mixed genetic background involving 129P2/OlaHsd × C57BL/6J (https://www.mmrrc.org/). All animal experiments were performed under protocols approved by the Animal Care and Experimentation Committee of Kyoto University.

### Behavioural activity monitoring

Single-caged adult male littermate mice (8- to 15-week old) were housed individually in light–tight, ventilated closets within a temperature- and humidity-controlled facility. The animals were entrained on a 12-h light (∼200 lux fluorescent light):12-h dark (LD) cycle for at least 2 weeks and then transferred to DD. Locomotor activity was detected with passive (pyroelectric) infrared sensors (FA-05 F5B; Omron) and the data were analysed with ClockLab software (Actimetrics) developed on MatLab (Mathworks)^29^. Free-running circadian period was determined with *χ*^2^ periodogram, based on animal behaviours in a 21-day interval taken 3 days after the start of DD condition. Fast Fourier transform (FFT) spectral analysis of the activity records was conducted with MATLAB Signal Processing Toolbox 6.2 (Mathworks). To extract long-term locomotor activity trends, we applied a moving average with a 3.67-h window size three times to the original locomotor activity data collected every 20 min. Then, FFT spectrograms were created through ‘specgram' command, with window size 512, overlap set to 506, and sampling set to 72 cycles per day. For light-pulse-induced shift experiments, mice put in DD were exposed to a 15-min light pulse at either CT14 or CT22. Phase shifts (delay at CT14, advance at CT22) were quantified as the time difference between regression lines of activity onset before and after the light application, using ClockLab software.

### *Per1-luc* organotypic tissue slice culture

*Per1-luc* transgenic mice carry a firefly luciferase reporter gene linked to a 7.2-kb genomic DNA fragment covering the 5′-upstream region of the mouse *Per1* gene^62^. *Per1-luc*-*Gpr176*^−/−^ mice were generated by crossing *Per1-luc* mice with *Gpr176*^−/−^ mice. The SCN slices were prepared according to our standard method^36^ and kept at 35 °C in a sealed 35 mm Petri dish with 1 ml of the culture medium containing 1 mM D-luciferin. Bioluminescence from the cultured SCN was measured with a highly sensitive cryogenic CCD camera (800S: Spectral Instruments) equipped with a microscope (Axiovert 200: Carl Zeiss). Recording was performed every 20 min. Observed data of images were filtered through a median filtre to eliminate cosmic-ray-induced background noise using ImageJ (http://imagej.nih.gov/ij). For period determination, the bioluminescence values of whole SCN in the image sequence were exported into Excel (Microsoft), where values were detrended by subtraction of baseline bioluminescence based on a running average from 12 h before to 12 h after each time point. Then, the baseline-subtracted data were curve fitted to a modified damped sine wave in Prism (Graphpad software) using the following equation: *Y*=Amplitude × exp(−*K* × *X*) × sin ((2 × *π* × *X*/Period)−Phase × 2 × *π*/Period), where *K* is the damping constant, and the period was determined based on the best-fit results. As for lung explant culture, culture was performed according to a published method^63^ with slight modifications. In brief, lungs taken from 5-day-old pups were sliced into a small piece (∼2 × 2 × 0.3 mm^3^), placed on a Millicell membrane (PICMORG50, Millipore) with 800 μl of DMEM medium (Sigma), supplemented with 10 mM HEPES (pH 7.2), 2% B27 (Invitrogen), 25 units ml^−1^ penicillin, 25 mg ml^−1^ streptomycin, and 1 mM luciferin, in 35-mm dish, and air sealed. Bioluminescence was continuously monitored without interruption for >5 d immediately upon placement in culture with a dish-type photon countable luminometer (Kronos Dio, ATTO) at 35 °C. Period of circadian luminescence was determined as described for the SCN.

### Radioisotopic *in situ* hybridization

*In situ* hybridization was performed with free-floating brain sections (30-μm thick), using [^33^P]-labelled cRNA probes for *Gpr176* (nucleotides 3321–3746, NM_201367), *Ntsr2* (1001–1480, NM_008747), *Gpr37l1* (1368–1849, NM_134438), *Lphn1* (7617–8072, NM_181039), *Oprl1* (1513–2006, NM_011012), *Gpr37* (2056–2528, NM_010338), *Gprc5b* (2269–2837, NM_022420), *Gpr85* (1942–2440, NM_145066), *Darc* (695–1103, NM_010045), *Lphn3* (5451–5771, NM_198702), *Lphn2* (5213–5697, NM_001081298), *Calcr* (670–1168, NM_007588), *Gpr48* (4320–4803, NM_172671), *Gpr56* (1844–2334, NM_018882), *Gpr19* (250–748, NM_008157), *Gpr123* (3955–4486, NM_177469), *Gpr83* (1518–2057, NM_010287), *Ackr3* (931–1392, NM_007722), *Gpr125* (3919–4377, NM_133911), *Gpr22* (2501–3002, NM_175191), *Gpr153* (3205–3689, NM_178406), *Gpr45* (1165–1722, NM_053107), *Gpr68* (2670–3170, NM_175493), *Gpr6* (1406–1747, AK139367), *Cckar* (2182–2593, NM_009827), and *Gpr75* (2269–2739, NM_175490). All fragments were sequenced to verify their identity, and antisense riboprobes were generated.

### Digoxigenin *in situ* hybridization

Digoxigenin *in situ* hybridization was performed according to our standard method^64^ with two different probes for *Gnaz* (NM_010311), a 5′UTR probe (296 bp, nucleotides 151–446) and a 3′UTR probe (232 bp, nucleotides 1,919–2,150), the sequences of which are divergent from those of the other Gi/o family members.

### Northern blotting

Northern blot analysis was performed with the following probes for *Gpr176* (NM_201367): 5′ probe (289 bp, nucleotides 132–420) and 3′ probe (426 bp, nucleotides 3,321–3,746). Both fragments were labelled with [^32^P] deoxycytidine triphosphate by random priming and hybridized with the mouse MTN blots (Clontech) to which poly(A)+ RNA fractions from various tissues were transferred (2 μg for each tissue).

### Microarray analysis

Microarray data have been deposited in the Gene Expression Omnibus under accession code GSE28574 (ref. 29). To identify genes that are enriched in the SCN, SCN punches taken from 10 animals at CT2 and 10 animals at CT14 were pooled together and analysed with a GeneChip Mouse Genome 430 2.0 (Affymetrix)^29^. The data were normalized with the MAS5 (GCOS 1.4) algorithm, using the default analysis settings and global scaling for normalization. For statistical analysis of the microarray data, we transformed the values into log2 format for ease of comparison and data representation. We then obtained values of 13 probes for the seminal proteins (Svs3, Svs5, Svs6, Sva, Svp2, Sval1 and Sval2) that were unlikely to be expressed in the SCN. We determined the mean and s.d. of these values. We took this mean value to represent zero expression and subtracted it from the value for each receptor for which we obtained a signal. The value of two s.d. was then considered to be the threshold or baseline for our receptors of interest as any values at this level or higher would be considered statistically significant (95% confidence that the true mean of the seminal protein expression would fall within this threshold value)^49^.

### Laser microdissection of the SCN

Coronal brain section (30-μm thick) containing the SCN was prepared using a cryostat microtome (CM3050S, Leica) and mounted on POL-membrane slides (Leica). Sections were fixed for 3 min in an ice-cold mixture of ethanol and acetic acid (19:1), rinsed briefly in ice-cold water, stained for 30 seconds in ice-cold water containing 0.05% toluidine blue, followed by two brief washes in ice-cold water. After wiping off excess water, slides were quickly air dried at room temperature. As soon as moistures in the sections decreased enough for laser-cutting, cells in the SCN were microdissected using a LMD7000 device (Leica) and lysed in Trizol reagent (Invitrogen), and total RNA was purified using the RNeasy micro kit (Qiagen).

### qRT–PCR

qRT–PCR analysis was performed as described previously with a standard curve method^65^,^66^. The data were normalized with *Rplp0*. The primer sets used were following: for mouse, *Gpr176* (NM_201367), Fw: 5′- CATCTTCATTGGCTCGCTAC -3′, Rv: 5′- CGTATAGATCCACCAGCAAC -3′; *Gnaz* (NM_010311), Fw: 5′- CAGCCGTGCTTAGAAACATCG -3′, Rv: 5′- TCTAGTGACACTCCACCTCC -3′; *Gnai1* (NM_010305), Fw: 5′- AAGCTGACTCGCCTTCCCAG -3′, Rv: 5′- GTAGTTTACAGTTCTCCACACG -3′; *Gnai2* (NM_008138), Fw: 5′- TGCCTTGAGTGTGTCTGCGTG -3′, Rv: 5′- CTCAGTGACGTTGGCAGTTG -3′; *Gnai3* (NM_010306), Fw: 5′- GTGCAGTCCGTGTACAAGAG -3′, Rv: 5′- GATGAATGGATCCGAGCCAC -3′; *Per1* (NM_011065), Fw: 5′- TGGCTCAAGTGGCAATGAGTC -3′, Rv: 5′- GGCTCGAGCTGACTGTTCACT -3′; and *Rplp0* (NM_007475), Fw: 5′- CTCACTGAGATTCGGGATATG -3′, Rv: 5′- CTCCCACCTTGTCTCCAGTC -3′; and for human, *GNAZ* (NM_002073), Fw: 5′- CTACGAGGATAACCAGAC -3′, Rv: 5′- TACGTGTTCTGGCCCTTG -3′; *GNAI1* (NM_002069), Fw: 5′- CATCTCTGACCTTGTTTCAGC -3′, Rv: 5′- CTTCAACCCAGTGACAACACG -3′; *GNAI2* (NM_002070), Fw: 5′- ACTCCGTGCCTTGAGTGTG -3′, Rv: 5′- TTGTCTGGAACAGCCCTTGG -3′; *GNAI3* (NM_010306), Fw: 5′- GGAAAGTTACGTTCACTTCAACC -3′, Rv: 5′- TTGGACCCCAAAAGGCACTG -3′; and *RPLP0* (NM_053275), Fw: 5′- ATGCAGCAGATCCGCATGT -3′, Rv: 5′- TTGCGCATCATGGTGTTCTT -3′.

### Antibodies to Gpr176 and Vipr2

Gpr176 antibody was raised in rabbit using a glutathione-*S*-transferase-fused Gpr176 mouse protein fragment (amino acids (a.a.) 311–515). The raised antibodies were affinity-purified using a maltose-binding protein (MBP)-fused Gpr176 fragment (a.a. 311–515). Vipr2 antibody was raised in chicken with a keyhole-limpet hemocyanin (KLH)-conjugated synthetic peptide mapping a C-terminal region of the mouse Vipr2 (a.a. 418−437). The antibodies were affinity-purified using the antigen peptide. Rabbit antiserum against Vipr2 was purchased from Abcam (ab28624).

### Immunohistochemistry

Free-floating immunohistochemistry was performed with 30-μm-thick serial coronal brain sections. To minimize technical variations in immunostaining, sections from different CTs were immunolabelled simultaneously. The primary antibodies used were anti-Gpr176 (final concentration, 0.6 μg ml^−1^), anti-Vipr2 rabbit antiserum (Abcam, 1:1,000), and anti-Vipr2 purified chicken polyclonal antibody (0.17 μg ml^−1^). Immunoreactivities were visualized with a peroxidase-based Vectorstain Elite ABC kit (Vector Laboratories) using diaminobenzidine chromogen. For brightness and contrast, photomicrographs were processed identically with ImageJ. For quantitative analysis, data were normalized with respect to the difference between signal intensities in equal areas of the SCN and the corpus callosum. Normalized values were summed from the rostral to the caudal margins of the SCN (10 sections per brain), and the sum was considered a measure for the amount of protein in the SCN. Values are expressed as means±s.e.m. (*n*=6, for each time point). For dual-label immunofluorescence, free-floating sections were stained with anti-Gpr176 (rabbit polyclonal, final concentration, 0.6 μg ml^−1^) together with either anti-Vipr2 (chicken polyclonal, 0.17 μg ml^−1^), anti-Vip (Abnova, guinea pig polyclonal, PAB16648, 1:1,000), or anti-Avp-associated neurophysin II (Santa Cruz, goat polyclonal, sc-27093, 0.2 μg ml^−1^) antibody. We visualized immunoreactivities using Alexa594-conjugated anti-rabbit IgG (1:1,000; Life Technologies) and Alexa488-conjugated anti-chicken, guinea pig, or goat IgG (1:1,000; Life Technologies). Nuclei were visualized by staining with 4′,6′-diamino-2-phenylindole (DAPI).

### Immunocytochemistry of dispersed SCN neurons

For colocalization analysis of Gpr176 and Vipr2, dissociated SCN neuronal cultures were used. SCN punches of 15 pups (C57Bl/6) of 4–5 days of age were pooled and incubated for 40 min at 37 °C in Ca^2+^/Mg^2+^-free Hanks' balanced salt solution (HBSS, Life Technologies) containing 0.06% papain, 0.02% L-cysteine, and 1 kU ml^−1^ DNaseI (Sigma). Following the addition of fetal bovine serum to the solution, cells were further dissociated through trituration with a fire-polished Pasteur pipette. The dispersals were then filtered through a 100-μm nylon cell strainer (BD Falcon) and resuspended in Neurobasal medium (Life Technologies) containing B27 supplement (Life Technologies) with 2 mM glutamine, 8 mM glutamate, 100 U ml^−1^ penicillin, and 100 μg ml^−1^ streptomycin. Cell viability was 85–95%. Viable cells were plated on polylysine-coated coverslips in 48-well culture plates at a density of 2,000 cells per mm^2^. One-half of the culture medium was exchanged with fresh medium every 3 days. After 8 days culture, cells were fixed with 4% paraformaldehyde and double-labelled for Gpr176 and Vipr2 using published methods^67^. We stained cultures with anti-Gpr176 (purified rabbit polyclonal, final concentration 0.15 μg ml^−1^) and anti-Vipr2 (purified chicken polyclonal, 0.15 μg ml^−1^) followed by Alexa594-conjugated antirabbit IgG (1:1,000; Life Technologies) and Alexa488-conjugated antichicken IgG (1:1,000; Life Technologies). Nuclei were visualized using DAPI staining.

### Stable cell lines

Flp-In TREx293-Gpr176 cells were generated by stable transfection of Flp-In T-Rex-293 cells (Life Technologies) with a pcDNA5/FRT vector (Life Technologies) containing the untagged full-length coding sequence of the mouse *Gpr176* (NM_201367). Similarly, Flp-In TREx293-Vipr2 cells were established with the mouse *Vipr2* full-length coding sequence (NM_009511). To develop Flp-In TREx293-Gpr176(tet-on)/Vipr2 cells, we constructed a modified pcDNA5/FRT vector carrying *Vipr2* and *Gpr176* under different promoters: while *Gpr176* was cloned into a proprietary pcDNA5/FRT cloning site for tet-on induction, *Vipr2* was cloned separately into a different position of the vector (at a unique *Pci*I site) in conjunction with a tetracycline-insensitive CMV promoter. Point mutations for Gpr176^RDY^ and Gpr176^V145R^ were introduced into the corresponding constructs with a standard sequential PCR method^68^. For stable expression of DN-Gz mutant (G204A/E246A/A327S)^52^, we established Flp-In TREx293-Gpr176(tet-on)/DN-Gz cells by using the modified pcDNA5/FRT vector as mentioned above. For infection of RGSZ1, lentiviruses carrying the hemagglutinin (HA)-tagged full-length coding sequence of RGSZ1 (NM_003702) in tandem with *IRES*-GFP (HA-RGSZ1-IRES-GFP) were generated with pCSII-EF-MCS-IRES-hrGFP vector^69^, and the cells were infected with an empirical titre of virus that resulted in nearly 100% infection as determined by GFP expression. Stable clonal NIH 3T3 cell lines expressing Gpr176 (NIH3T3 Tet-on 3G-Gpr176) were generated via transfection of NIH3T3 Tet-On 3G cells (Clontech) with a pTRE3G vector (Clontech) containing *Gpr176*. The established cells were further transfected with a pEF vector (Addgene) containing *Gz* (NM_010311) along with Linear Puromycin Marker (Clontech) to generate NIH3T3 Tet-on 3G-Gpr176(tet-on)/Gz double stable cell clones. Cells were cultured in DMEM containing 10% fetal bovine serum with an appropriate mixture of antibiotics that the manufacturers recommend for the maintenance of the cell clones.

### Immunoblot

To avoid high-temperature-induced protein aggregation of GPCR, cell lysates were denatured on ice in Laemmli buffer and subjected to SDS–PAGE at 4 °C. Immunoblotting was performed using our standard method^29^ with affinity-purified antibodies against Gpr176 (rabbit polyclonal, final concentration, 0.6 μg ml^−1^) and Vipr2 (chicken polyclonal, 0.4 μg ml^−1^). To detect Gz, the plasma membrane fractions were lysed in standard RIPA buffer and immunoprecipitated with anti-Gz antibody (Santa Cruz, sc-388, 1 μg per immunoprecipitaion), and immunoblots were probed with the rabbit antisera against Gz (2919) provided by Dr Manning^48^,^70^ (1:100 dilution). Commercially available antibodies against Gi (Abcam, ab3522, 1 μg ml^−1^) and α-tubulin (Sigma, T6199, 1 μg ml^−1^) were used as a control. Blot images have been cropped for presentation. Full size images are presented in Supplementary Fig. 12.

### Measurements of cAMP and IP_1_

After 24 h of treatment with Dox (1 μg ml^−1^) or vehicle, cells were removed from culture dish with Versene solution (Life Technologies) and dissociated into single cells through gentle trituration. After filtration with a 100-μm cell strainer (BD Falcon), cells were resuspended in HBSS containing 5 mM HEPES (pH 7.5), 0.1% bovine serum albumin, and 0.5 mM 3-isobutyl-1-methylxanthine (IBMX, a non-selective phosphodiesterase inhibitor). Cells in suspension were incubated at 37 °C for 1 h in 24-well plates at a density of 3 × 10^5^ cells per well, followed by stimulation with forskolin (Nacalai Tesque) or Vip (Peptide Institute Inc.) at the indicated concentrations for 15 min. The reactions were stopped by adding ice-cold perchloric acid (1 N, final solution) containing 4 mM theophylline (Sigma). After 1 h at 4 °C, the mixtures were centrifuged, and the supernatants were neutralized with ice-cold 0.72 M KOH/0.6 M KHCO_3_. Following removal of salt precipitants, the extracts were assayed for cAMP concentrations with a cAMP-specific enzyme immunoassay kit (Cayman Chemical)^29^. Assays with PTX-treated cells were also done with the same protocols, except that the cells were cultured in the presence of PTX (100 ng ml^−1^, Bio Academia) for 16 h before assay. When specified, 100 μM sphingosine-1-phosphate (Enzo Life Sciences) was added together with forskolin, to confirm whether Gi-mediated signalling was blocked efficiently by the PTX treatment. For IP_1_ assay, cells in suspension were incubated in IP stimulation buffer (Cisbio; 10 mM HEPES (pH 7.4), 1 mM CaCl_2_, 0.5 mM MgCl_2_, 4.2 mM KCl, 146 mM NaCl, 5.5 mM glucose and 50 mM LiCl) for 1 h at 37 °C in 24-well plate at a density of 4 × 10^5^ cells per well. The reaction was stopped by adding Lysis reagent (Cisbio). The concentrations of IP_1_ were determined by enzyme immunoassay with IP-One ELISA kit (Cisbio) according to the manufacturer's protocol.

### [^125^I]-Vip binding assay

To prepare membrane fractions, Flp-In TREx293-Gpr176(tet-on)/Vipr2 cells were lysed in hypotonic lysis buffer (25 mM HEPES, pH 7.5, 1 mM EDTA, 1 mM DTT) with protease inhibitors, and passed through a 27-gauge needle 10 times. The lysed cells were centrifuged at 700 *g* for 5 min to remove nuclei and debris. The supernatant was centrifuged at 20,400*g* for 30 min. Then, the pellet (membrane fraction) was resuspended in HBSS containing 5 mM HEPES (pH 7.5). For the binding reaction, the membranes (10 μg of protein) were incubated with 1, 10 or 100 nM of [^125^I]-labelled Vip (PerkinElmer; NEX192) in HBSS binding buffer containing 5 mM HEPES (pH 7.5) and 0.1% bovine serum albumin for 60 min at 4 °C. Incubation was performed in a 1.5-ml siliconized tube (Sarstedt). At the termination of incubation, membrane-bound [^125^I]-Vip was separated from free peptide by centrifugation. The membrane pellets were washed three times with HBSS/5 mM HEPES/0.1% bovine serum albumin, and the washed membranes were assayed for [^125^I] radioactivity with a 1470 automatic gamma counter (PerkinElmer). Specific binding was calculated by subtracting the radioactivity detected for un-transfected (parental) Flp-In TREx293 cells.

### siRNAs

To knockdown Gz, two independent pools of Gz-specific silencer select siRNAs (#1 and #2), each containing three different siRNA duplexes directed against the Gz coding sequence (#1: s5898, s5900 and s499632; #2: s499631, s500931 and s500937; Life Technologies), were introduced into ∼30% confluent Flp-In TREx293-Gpr176 cells using Lipofectamine 2000 (Life Technologies) according to the manufacturer's instructions. As a control, we also transfected the cells with negative-control siRNA (catalog number 4390846, Life Technologies) at the same concentration as the #1 and #2 mixtures (1.8 nmol siRNA per 10-cm dish). Medium was replaced 6 h after transfection. Four days later, cells were treated with Dox (1 μg ml^−1^) or vehicle for 24 h and removed from dishes with Versene solution (Life Technologies) to be subject to cAMP assays. For each experiment, we took a fraction of cells and confirmed by qRT–PCR that Gz mRNA accumulation was diminished to <5% by RNA interference.

### SCN punch for cAMP measurement

The microdissection of the SCN was performed as described with modifications. Animals kept in DD were killed by cervical dislocation, and the eyes were removed under a safety red light. The brain was then isolated from the skull under room light and frozen immediately on dry ice. Coronal brain section (300-μm thick) containing the SCN was prepared using a cryostat microtome (CM3050S, Leica) and mounted on a silicon rubber stage at −17 °C. Under a magnifying glass, the bilateral SCN was punched out from the frozen section using a blunt 20-gauge syringe needle whose edge had been sharpened by filing. The microdissected SCN (one punch per assay) was then immediately sonicated at 4 °C (Bioruptor, COSMO BIO) in 0.1 N HCl solution containing 0.5 mM IBMX. Lysates were clarified by centrifugation, and the protein content was determined with a Bradford assay (Nakarai). The amount of extracted cAMP was measured using an enzyme immunoassay kit for cAMP (Cayman Chemical). To increase the sensitivity of the assay, samples were acetylated according to the manufacturer's instruction (Cayman Chemical).

### GloSensor cAMP assay

We generated additional Gpr176 mutants on the DRY triplet sequence (Supplementary Fig. 8; DAY, AAY, and AAA) and tested their basal activities using the cAMP GloSensor system (Promega), which allows transient transfection-based GPCR assay for cAMP signal. Flp-In TREx293 cells were plated on 35-mm dishes at 1.2 × 10^6^ cells per dish with a CO_2_-independent DMEM medium (Sigma) containing 10 mM HEPES (pH 7.2), 10% FBS, 100 units ml^−1^ penicillin, 100 mg ml^−1^ streptomycin at 37 °C. In the next day, the cells were transfected with a DNA mixture containing 1 μg of pGloSensor-22F plasmid (Promega) and 1.5 μg of pcDNA3.1 expression plasmid (Life Technologies) encoding either the untagged wild-type Gpr176 or its respective DRY mutants, using Lipofectamine LTX/Plus reagent (Life Technologies). Four hours after the transfection, the medium was refreshed to the medium with 1 mM luciferin, and the cells were further cultured at 37 °C for 14 h. Following 2-h incubation at 28 °C for equilibrium, GloSensor activities in the cells were measured using a dish-type luminometer (Kronos Dio, ATTO) at 28 °C. Recording was performed every 1 min with 2 s of integration. After detection of baseline luminescence activities for 10 min, Fsk was added to the culture medium at the final concentration of 10 μM. Recording was stopped at 30 min after the Fsk stimulation. Then the cells were immediately lysed into Laemmli buffer for western blot analysis.

## Additional information

**How to cite this article:** Doi, M. *et al*. Gpr176 is a Gz-linked orphan G-protein-coupled receptor that sets the pace of circadian behaviour. *Nat. Commun.* 7:10583 doi: 10.1038/ncomms10583 (2016).

## Supplementary Material

Supplementary Information Supplementary Figures 1-12
