# Supplementary material for: Gpr176 is a Gz-linked orphan G-protein-coupled receptor that sets the pace of circadian behaviour
Source: Nat Commun. 2016 Feb 17;7:10583. doi: 10.1038/ncomms10583 (PMC4757782; doi:10.1038/ncomms10583)
Supplement: Supplementary Information — Supplementary Figures 1-12 [file ncomms10583-s1.pdf]

Supplementary Figure 1

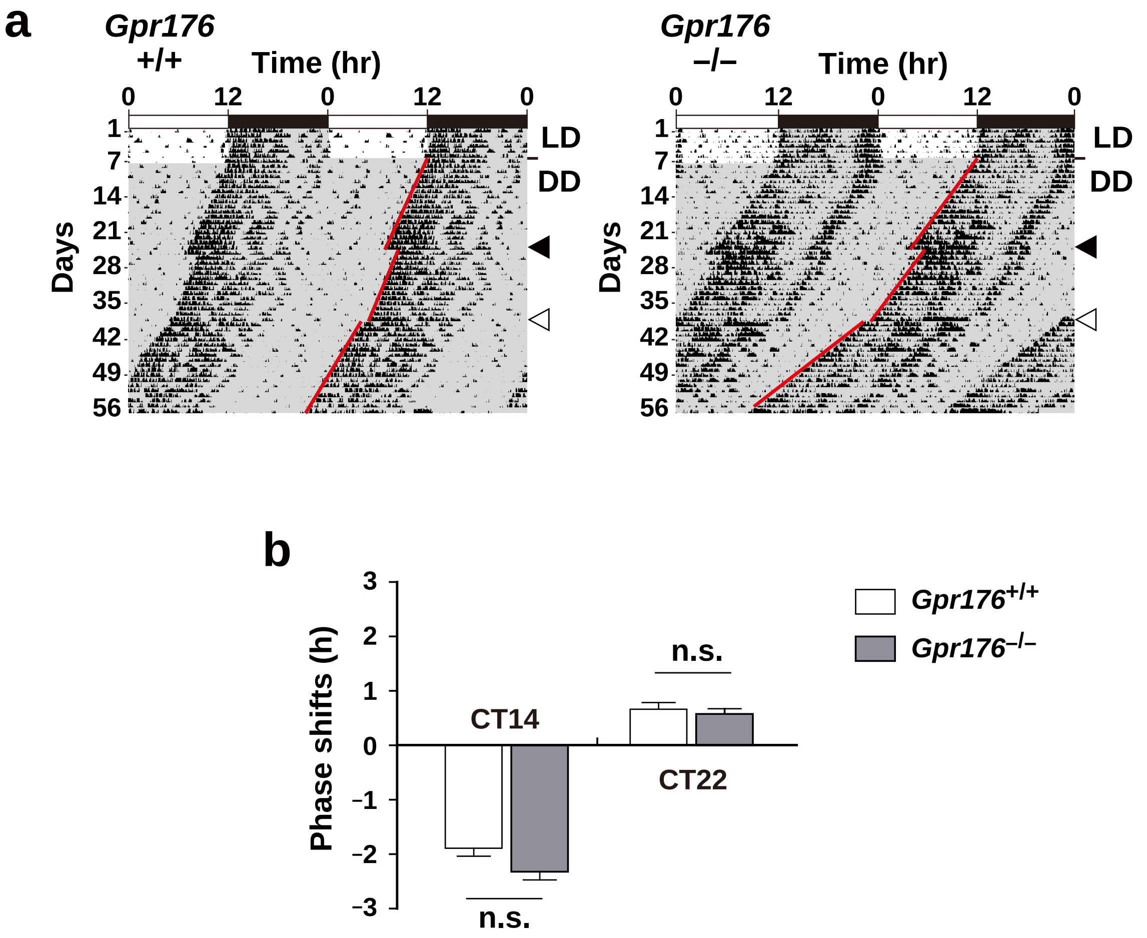

Supplementary Figure 1 Normal light ressetting responses of *Gpr176*$^{-/-}$ mice. (**a**) Representative double-plotted locomotor activity records of *Gpr176*$^{+/+}$ and *Gpr176*$^{-/-}$ mice. Mice initially housed in a 12:12 LD cycle were transferred to DD. A brief light pulse (200 lux light, for 15 min) was given at CT14 (on the day indicated by solid arrowheads) and CT22 (open arrowheads), which causes a phase delay and advance, respectively, on the following days. Red lines delineate the phase of activity onset. (**b**) Magnitude of light pulse-induced phase-shifts of *Gpr176*$^{+/+}$ (n = 8) and *Gpr176*$^{-/-}$ (n = 9) mice. By convention, delays are negative and advances are positive. Note that the wildtype and mutant mice are virtually the same in the extent of the phase shifts.

Supplementary Figure 2

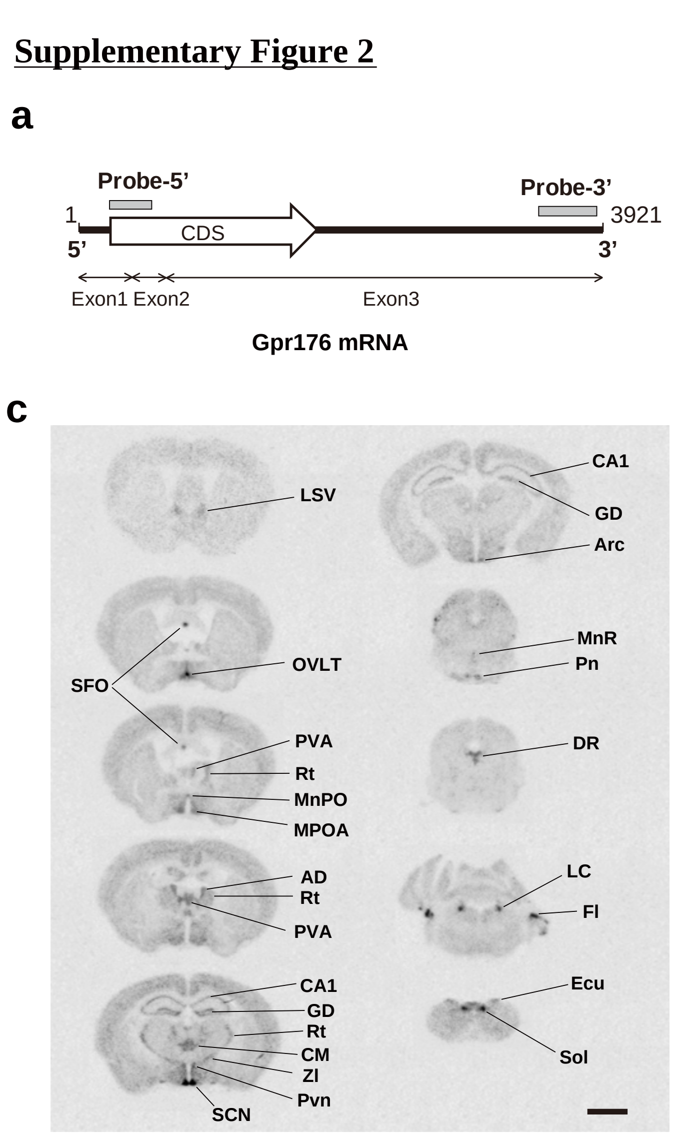

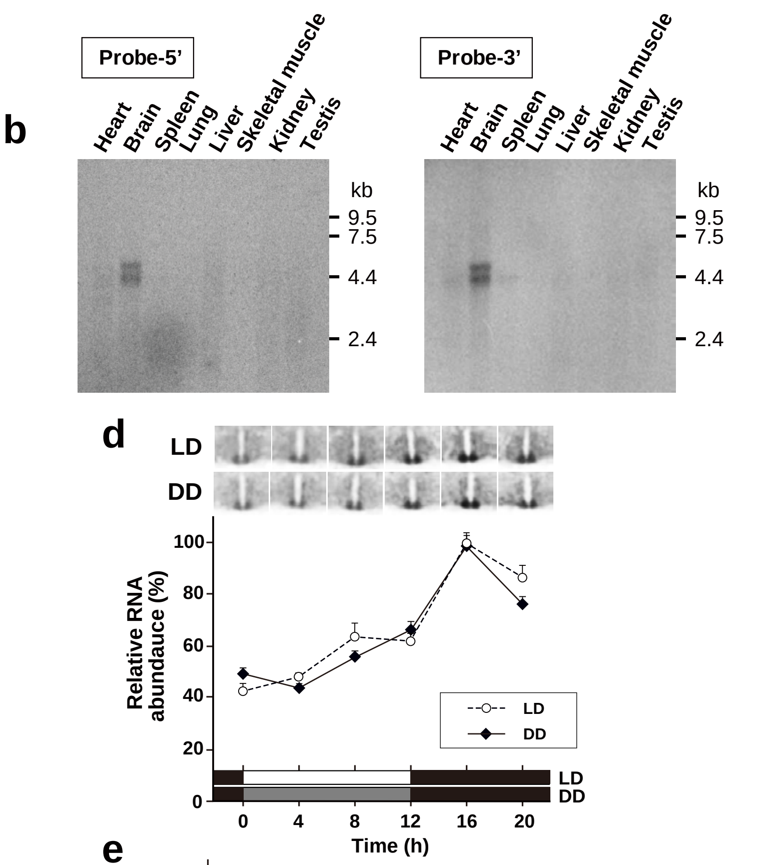

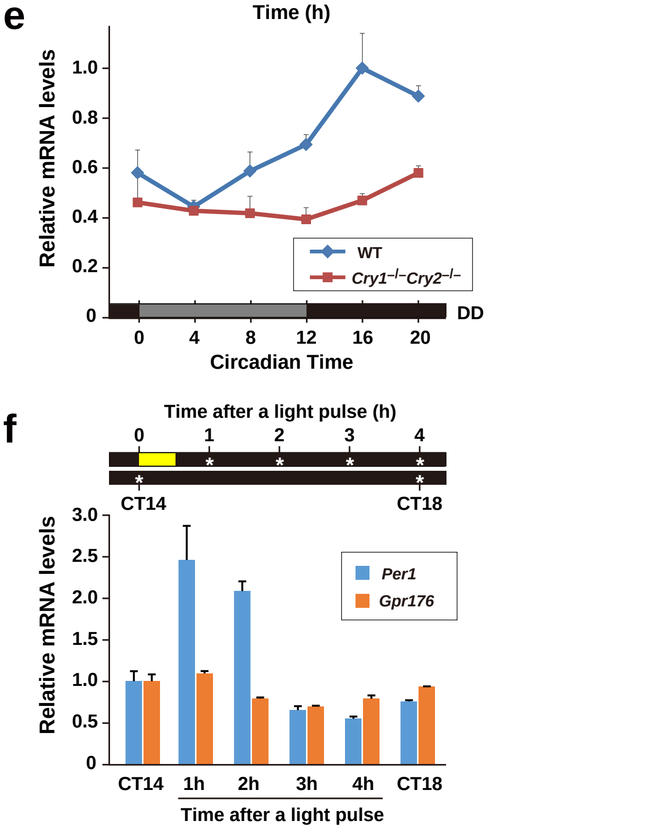

Supplementary Figure 2 Characterization of tissue distribution and brain mapping reveal prominent expression of *Gpr176* transcript within the SCN: *Gpr176* shows clock-controlled cyclic expression in the SCN. (**a**) Schematic structure of the mouse *Gpr176* transcript. CDS, coding sequence. (**b**) Northern blots probed for *Gpr176* using a 5'-portion of the coding region (5' probe) and a 3'-portion of the untranslated region (3' probe). Both of these probes illustrated that the *Gpr176* mRNA is expressed mainly in the brain. The positions of the RNA size markers are shown at the right. The blot for the 3' probe is reproduced from **Fig. 1c** for comparison. (**c**) Distribution of *Gpr176* in the mouse brain. Expression of *Gpr176* was examined in coronal sections from the forebrain to the medulla oblongata by *in situ* hybridization with isotope-labeled probe. Note that the SCN is a prominent site of *Gpr176* expression in the brain. AD, anterodorsal thalamic nucleus; Arc, hypothalamic arcuate nucleus; CA1, hippocampal CA1 subfield; CM, central medial thalamic nucleus; DR, dorsal raphe nucleus; Ecu, external cuneate nucleus; Fl, flocculus; GD, gyrus dentatus; LC, locus coeruleus; LSV, ventral part of lateral septal nucleus; MnPO, median preoptic nucleus; MnR, median raphe nucleus; MPOA, medial preoptic area; OVLT, organum vasculosum lamina terminalis; Pn, pontine nucleus; PVA, anterior paraventricular thalamic nucleus; Pvn, hypothalamic paraventricular nucleus; Rt, thalamic reticular nucleus; SCN, suprachiasmatic nucleus; SFO, subfornical organ; Sol, nucleus of the solitary tract; ZI, zona incerta. Scale bar, 2 mm. (**d**) Time-of-day-dependent changes in expression of *Gpr176* in the SCN under LD and DD conditions. Mice were sacrificed every four hours over a 24-h cycle in LD and DD. Relative RNA abundance in the SCN was quantified with radioisotopic *in situ* hybridization. Values are expressed as means ± s.e.m ($n = 5$) with the peak value adjusted to 100. Representative autoradiographs at the indicated time points are shown on the top. Note that *Gpr176* shows cyclic expression that peaks at night in both LD and DD. (**e**) Expression profiles of *Gpr176* in the SCN of wildtype (WT) and circadian clock-deficient $Cry1^{-/-}Cry2^{-/-}$ mice in DD. The SCN was dissected out by laser microdissection, and relative mRNA levels of *Gpr176* were determined by qRT-PCR and normalized to those of the ribosomal phosphoprotein P0 (Rplp0)-encoding gene. Values are plotted as means ± s.e.m ($n = 4$ mice for each data point) relative to the peak WT value. (**f**) Expression profiles of *Gpr176* and *Per1* in the SCN after a light stimulus given at CT14. C57BL/6 mice were exposed to light (200 lux fluorescent light) at CT14 for 30 min and sacrificed 1, 2, 3, 4 h after the start of the light exposure. No-light-exposed animals at CT14 and CT18 served as control groups. Relative mRNA levels of *Gpr176* and *Per1* in the SCN were determined as described in (**e**). Note that the light stimulus increased expression of *Per1* but not that of *Gpr176*.

Supplementary Figure 3

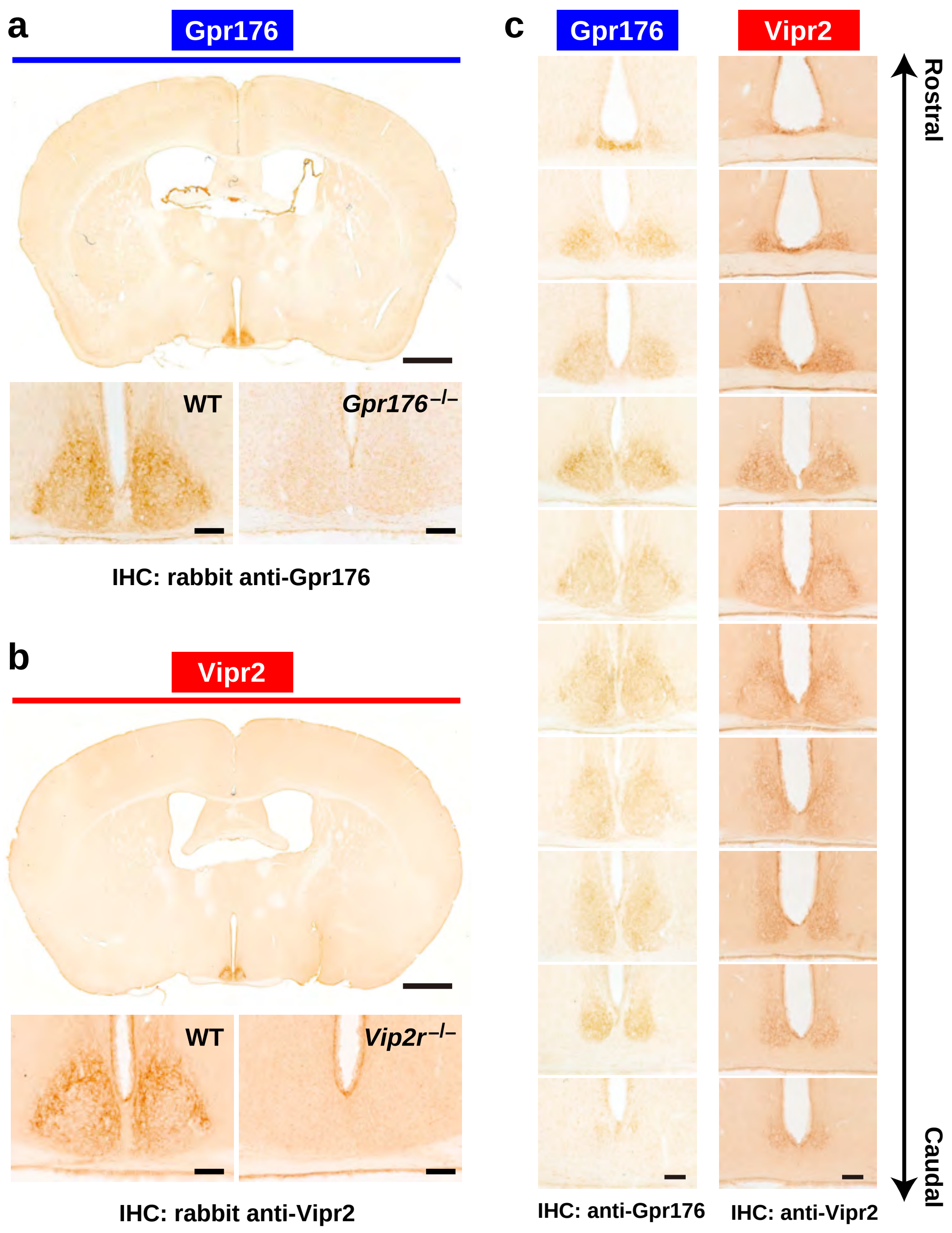

Supplementary Figure 3 Immunohistological distributions of Gpr176 and Vipr2 are similar in the SCN. (**a**) Immunolocalization of Gpr176 in the mouse SCN. (Upper) A representative coronal brain section immunolabeled for Gpr176, related to **Fig. 1d**. Scale bar, 1 mm. (Lower) High-power photomicrographs of the SCN sections from wildtype (WT) and *Gpr176*-deficient (*Gpr176*$^{-/-}$) mice, related to **Fig. 1g**. Scale bar, 100 µm. Rabbit anti-Gpr176 antibody was used for immunohistochemistry (IHC). (**b**) Immunolocalization of Vipr2 in the mouse SCN. (Upper) A representative coronal brain section immunolabeled for Vipr2, using rabbit anti-Vipr2 antibody. Scale bar, 1 mm. (Lower) High-power photomicrographs of the SCN sections from WT and *Vipr2*-deficient (*Vipr2*$^{-/-}$) mice. Scale bar, 100 µm. Reduced immunostaining in *Vipr2*$^{-/-}$ confirms the specificity of the antibody used. Both Gpr176 and Vipr2 are enriched in the SCN. (**c**) Immunohistological distributions of Gpr176 (left) and Vipr2 (right) in coronal sections from rostral to caudal margins of the mouse SCN. Scale bar, 100 µm. The shape of the SCN in the coronal plane differs when sections are lined from the rostral to caudal sides (ref*). At the rostral level, the SCN is flat just above the optic chiasm. At the middle level, the SCN is elliptic, longer in the dorsoventral plane than the mediolateral plane. At the caudal level, SCN is severely elongated in the dorsoventral axis and very thin along the nearby third ventricle. Note that immunohistological distribution profiles of Gpr176 (left) and Vipr2 (right) are similar: they are both expressed from the rostral (top) to caudal (bottom) extremities of the SCN, and they tend to be more intense in the dorsomedial than the ventrolateral SCN.

ref*: Lydic, R., Albers, H.E., Tepper, B. & Moore-Ede, M.C. Three-dimensional structure of the mammalian suprachiasmatic nuclei: a comparative study of five species. *J Comp Neurol* **204**, 225-237 (1982).

Supplementary Figure 4

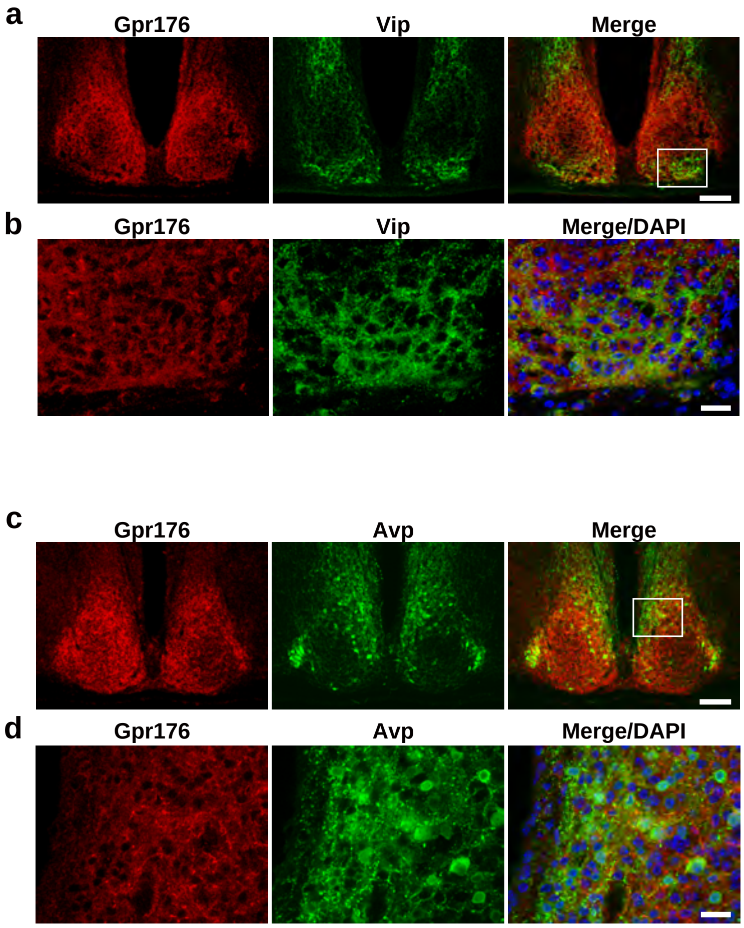

Supplementary Figure 4 Immunolocalization of Gpr176, Vip, and Avp in the mouse SCN.
(**a**) Double-label confocal immunofluorescence of Gpr176 and Vip. Coronal brain sections were immunolabeled with antibodies against Gpr176 (rabbit polyclonal) and Vip (guinea pig polyclonal). Representative confocal pictures are shown, with a merged image of Gpr176 (red) and Vip (green). Scale bar, 100 μm.
(**b**) Enlargement of the boxed area in (**a**). Merge shows combined images for Gpr176 (red), Vip (green), and DAPI-based nuclear staining (blue). Scale bar, 20 μm.
(**c**) Double-label confocal immunofluorescence of Gpr176 and Avp. Coronal brain sections were immunolabeled with antibodies against Gpr176 (rabbit polyclonal) and Avp (goat polyclonal). Representative confocal pictures are shown, with a merged image of Gpr176 (red) and Avp (green). Scale bar, 100 μm.
(**d**) Enlargement of the boxed area in (**c**). Merge shows combined images for Gpr176 (red), Avp (green), and DAPI-based nuclear staining (blue). Scale bar, 20 μm.

Supplementary Figure 5

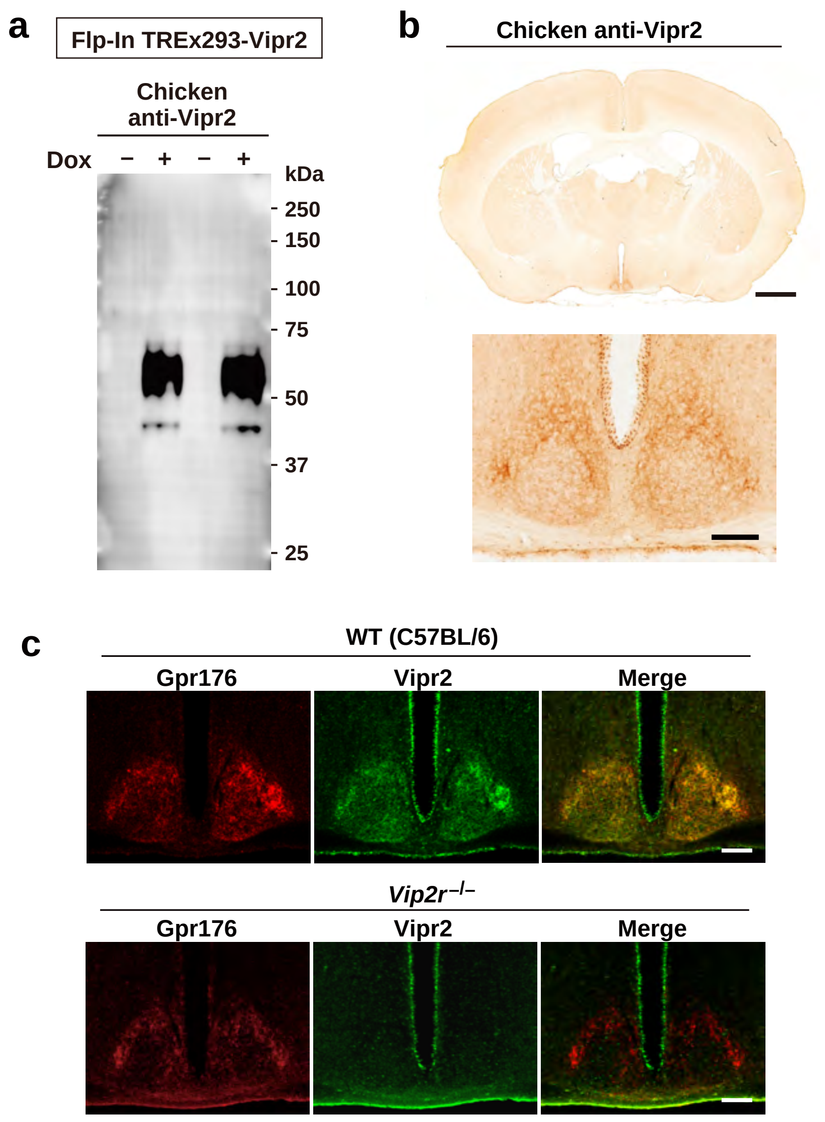

Supplementary Figure 5 Characterization of chicken polyclonal anti-Vipr2 antibody (**a**) Western blots with chicken polyclonal antibody against Vipr2. This antibody successfully detects Dox-induced Vipr2 protein in Flp-In TREx293-Vipr2 cells. (**b**) A representative image of the mouse coronal brain section immunolabeled with chicken anti-Vipr2 antibody, with an enlarged view of the region containing the SCN. Scale bars, 1 mm for the upper panel, and 100 µm for the lower panel. (**c**) Dual-label immunostaining of Gpr176 (rabbit polyclonal) and Vipr2 (chicken polyclonal) with the mouse coronal SCN sections from wildtype (C57BL/6) and *Vipr2*-null mutant mice (*Vipr2*$^{-/-}$). The images for the wildtype mice are reproduced from **Fig. 2a**. Note that the sections from *Vipr2*$^{-/-}$ mice had decreased Vipr2-immunoreactivities in the SCN. Non-specific reactions were observed on the epithelial cells that surround the third ventricle. Scale bar, 100 µm.

Supplementary Figure 6

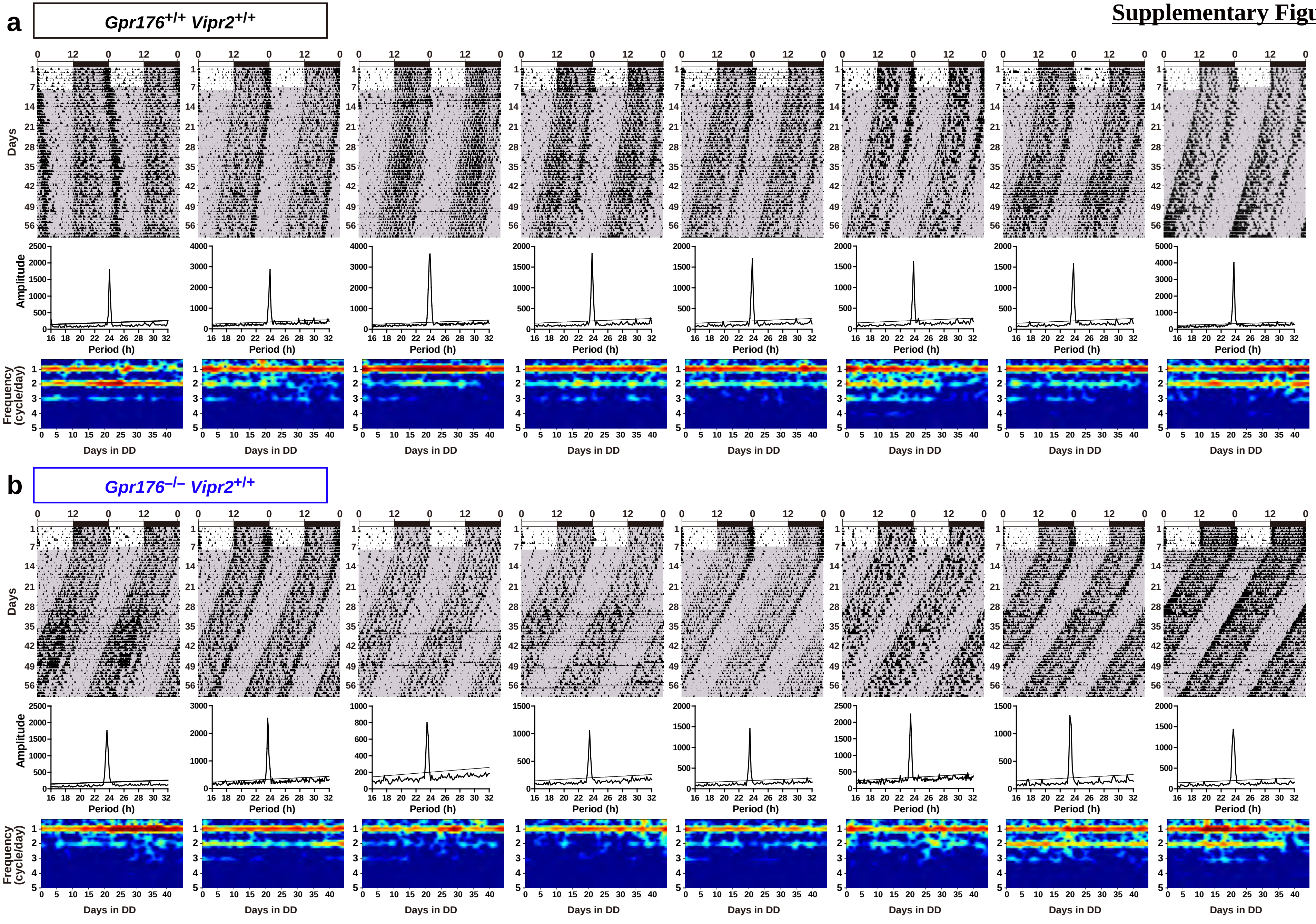

Supplementary Figure 6 Circadian behavioral data of all individual mice tested in **Fig. 2e–g**. Each panel shows double-plotted actograms (upper), chi-square periodograms (middle), and fast Fourier transform (FFT) spectrograms (lower) of locomotor activity rhythms of mice with the following features: (**a**,**b**) Mice with or without *Gpr176* in *Vipr2*$^{+/+}$ genetic background. (**c**,**d**) Mice with or without *Gpr176* in *Vipr2*$^{-/-}$ genetic background, showing a single dominant circadian period in DD. (**e**,**f**) Mice with or without *Gpr176* in *Vipr2*$^{-/-}$ genetic background, showing multiple circadian periods in DD. Mice were initially held in LD and transferred to DD after 8 d. The straight diagonal lines on periodograms indicate significance at $P < 0.001$. FFT spectrograms of the records show a consistent frequency (about one cycle per day) for wildtype (**a**) and *Gpr176* single knockout (**b**) mice; however, more variable (or intermittent) frequency components were observed for the mice carrying the *Vipr2*$^{-/-}$ allele (**c**–**f**).

Supplementary Figure 6

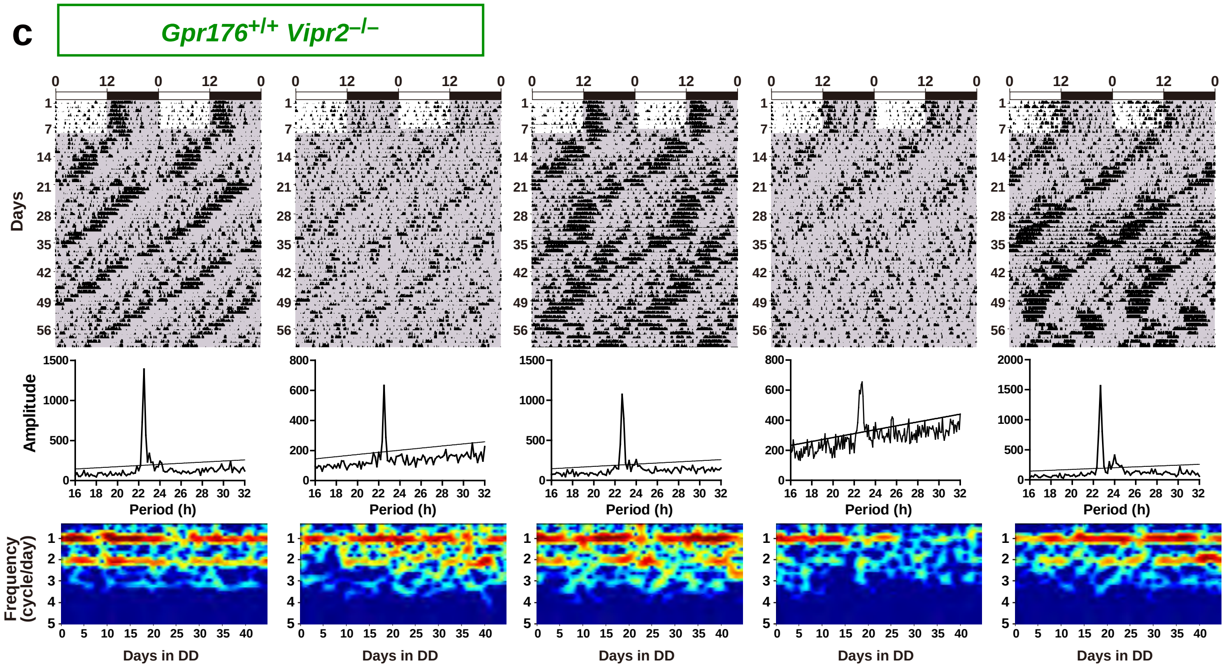

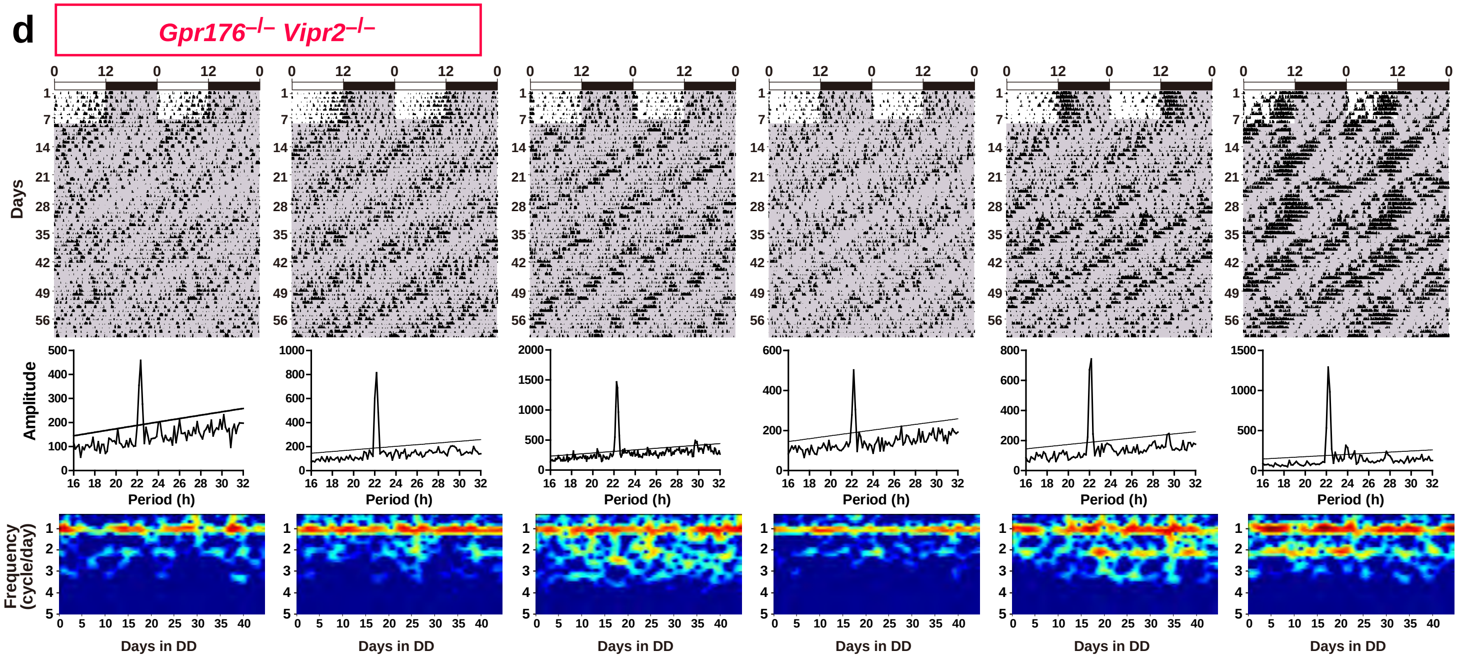

Supplementary Figure 6 Circadian behavioral data of all individual mice tested in **Fig. 2e–g**. Each panel shows double-plotted actograms (upper), chi-square periodograms (middle), and fast Fourier transform (FFT) spectrograms (lower) of locomotor activity rhythms of mice with the following features: (**a**,**b**) Mice with or without *Gpr176* in *Vipr2*$^{+/+}$ genetic background. (**c**,**d**) Mice with or without *Gpr176* in *Vipr2*$^{-/-}$ genetic background, showing a single dominant circadian period in DD. (**e**,**f**) Mice with or without *Gpr176* in *Vipr2*$^{-/-}$ genetic background, showing multiple circadian periods in DD. Mice were initially held in LD and transferred to DD after 8 d. The straight diagonal lines on periodograms indicate significance at $P < 0.001$. FFT spectrograms of the records show a consistent frequency (about one cycle per day) for wildtype (**a**) and *Gpr176* single knockout (**b**) mice; however, more variable (or intermittent) frequency components were observed for the mice carrying the *Vipr2*$^{-/-}$ allele (**c**–**f**).

Supplementary Figure 6

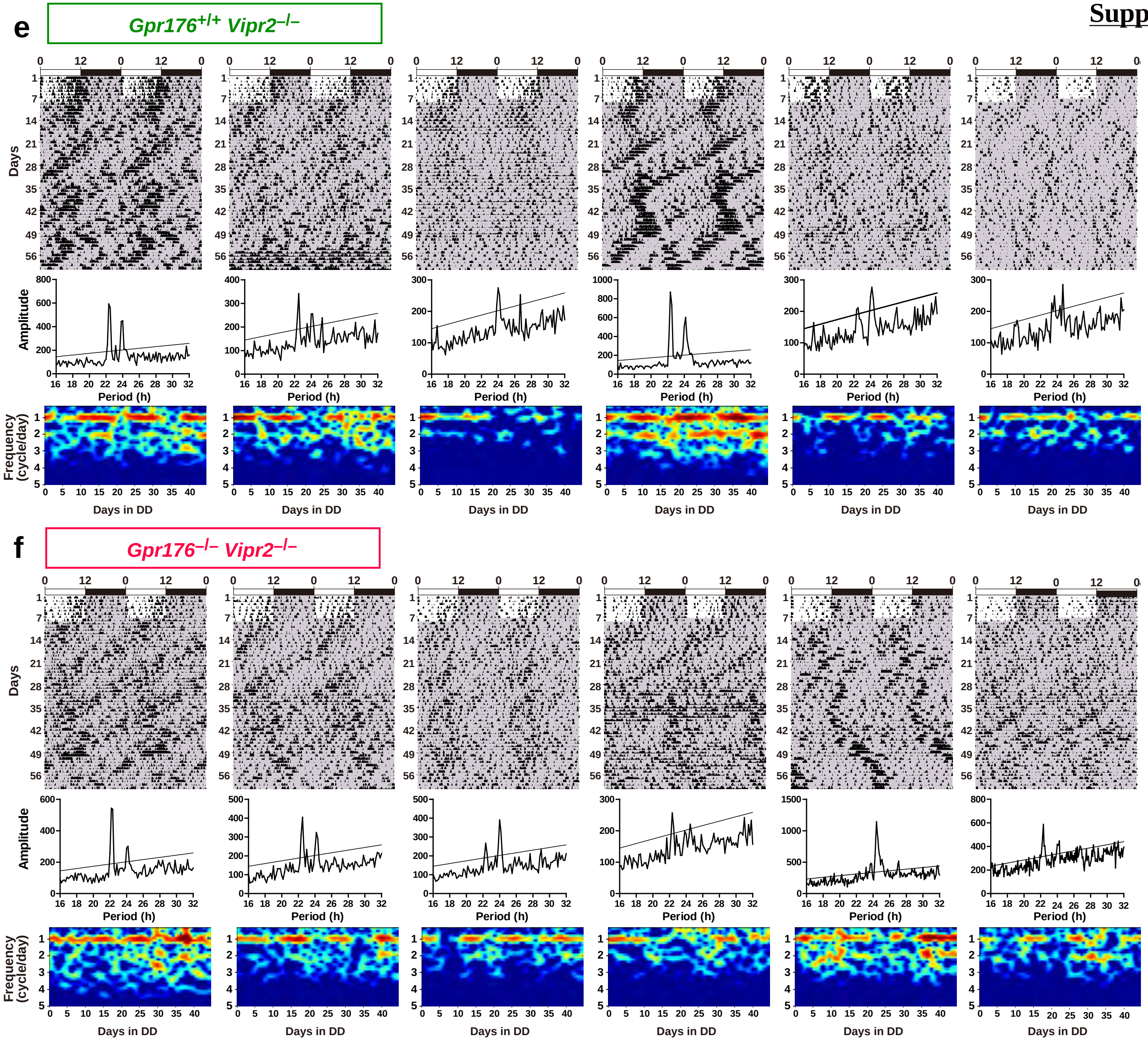

Supplementary Figure 6 Circadian behavioral data of all individual mice tested in **Fig. 2e–g**. Each panel shows double-plotted actograms (upper), chi-square periodograms (middle), and fast Fourier transform (FFT) spectrograms (lower) of locomotor activity rhythms of mice with the following features: (**a**,**b**) Mice with or without *Gpr176* in *Vipr2*$^{+/+}$ genetic background. (**c**,**d**) Mice with or without *Gpr176* in *Vipr2*$^{-/-}$ genetic background, showing a single dominant circadian period in DD. (**e**,**f**) Mice with or without *Gpr176* in *Vipr2*$^{-/-}$ genetic background, showing multiple circadian periods in DD. Mice were initially held in LD and transferred to DD after 8 d. The straight diagonal lines on periodograms indicate significance at $P < 0.001$. FFT spectrograms of the records show a consistent frequency (about one cycle per day) for wildtype (**a**) and *Gpr176* single knockout (**b**) mice; however, more variable (or intermittent) frequency components were observed for the mice carrying the *Vipr2*$^{-/-}$ allele (**c**–**f**).

Supplementary Figure 7

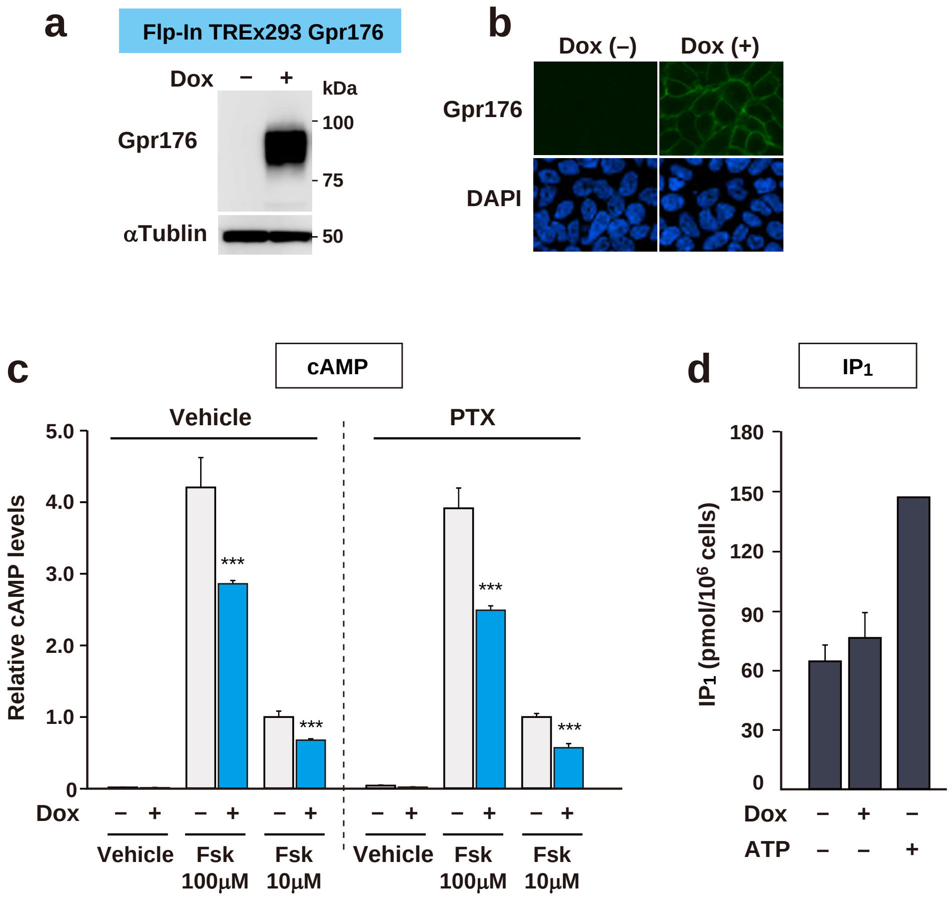

Supplementary Figure 7 Gpr176 basal activity reduces cAMP without affecting IP_1 accumulation, as demonstrated by Flp-In TREx293 cells bearing only Gpr176 (Flp-In TREx293-Gpr176). (**a**) Immunoblotting with Flp-In TREx293-Gpr176 cells. Cells were treated with Dox or vehicle for 24 h, and whole cell lysates were immunoblotted for Gpr176 and αTublin. (**b**) Representative confocal immunofluorescent images for Gpr176 in Dox-treated or nontreated Flp-In TREx293-Gpr176 cells. DAPI staining identifies all cell nuclei. (**c**) Antagonistic basal activities of Gpr176 on Fsk-stimulated cAMP accumulation in Flp-In TREx293-Gpr176 cells. Dox-treated or nontreated cells were resuspended in IBMX-containing assay buffer for 1 h and subjected to Fsk stimulation for 15 min. Where specified, cells were treated with PTX for 16 h before assay. cAMP values are plotted relative to those of Fsk-treated uninduced cells. Data are the mean ± s.e.m. of at least two independent experiments with three replicates each. ***$P < 0.001$ in comparison with the control stimulated group. (**d**) Unaffected IP_1 accumulation in Dox-treated Flp-In TREx293-Gpr176 cells. Cells were treated with or without Dox for 24 h and resuspended in IP stimulation buffer (see **Methods**) for 1 h. IP_1 levels were determined by enzyme immunoassays. As a positive control, we used ATP stimulation (50 µM, 1 h), which induces Gq signaling in TREx293 cells. Values are expressed as mean ± s.e.m. per 1×10^6 cells of three independent experiments.

Supplementary Figure 8

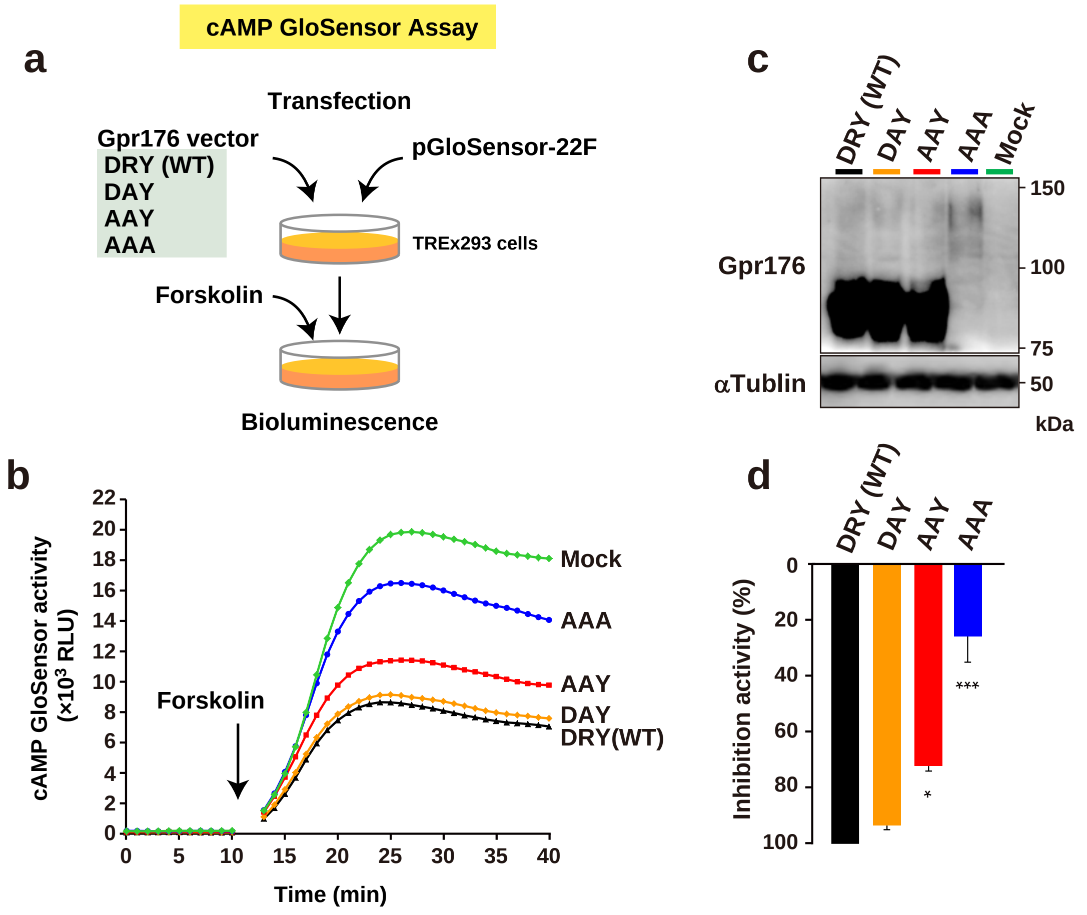

Supplementary Figure 8 Effects of the DRY motif mutations on the levels of protein expression and cAMP-repressing activities of Gpr176. (**a**) Schematic of experimental design. We made three different mutants, i.e. D**A**Y, **AA**Y, and **AAA**, and assessed their basal activities by using the cAMP GloSensor system (Promega), which allows transient transfection-based GPCR assay for cAMP signal. (**b**) Fsk-induced cAMP GloSensor luciferase activities in TREx293 cells transfected without (Mock) or with respective Gpr176 expression constructs. Luminescence was measured in cell culture at 1 min intervals. The arrow indicates 10 μM Fsk treatment. RLU, relative light units. (**c**) Representative immunoblot image for wildtype (WT) Gpr176 and respective mutants. Cells were lysed immediately after the measurement of luminescence and immunoblotted for Gpr176 and αTublin. (**d**) Percent cAMP inhibition activities of the mutants, relative to WT control. Luminescence values from 3 to 30 min post Fsk treatment were integrated and divided by those of the Mock transfection control. The repressing activities of the mutants are shown as relative percent to WT (100%). Data are the means ± s.e.m. of three independent experiments. ***$P < 0.001$, *$P < 0.05$, vs. WT, one-way ANOVA with Bonferroni *post-hoc* test. Note that transfection of WT Gpr176 resulted in lowered luminescence (thus, decreased cAMP accumulation) after Fsk treatment, as compared to mock transfection control (compare green vs. black in (**b**)). Under these conditions, the D**A**Y mutant was almost intact for both the activity and protein expression levels (see orange in (**b**), (**c**), and (**d**)). In contrast, the cAMP repressing activities of the **AA**Y and **AAA** mutants were significantly reduced to about 70% and 30% relative to WT values, respectively (see red and blue in (**b**) and (**d**)). Importantly, expression levels of the **AA**Y mutant were comparable to those of WT (see (**c**)), thus suggesting that this mutation leads to decreased basal activities without affecting protein levels. On the other hand, the **AAA** mutation caused a drastic loss of the protein with a concomitant generation of slowly migrating bands (see around 130 kDa in (**c**)), suggesting a fundamental protein structure problem. These data support our argument that the DRY motif is important for both the protein activity and stability of Gpr176.

Supplementary Figure 9

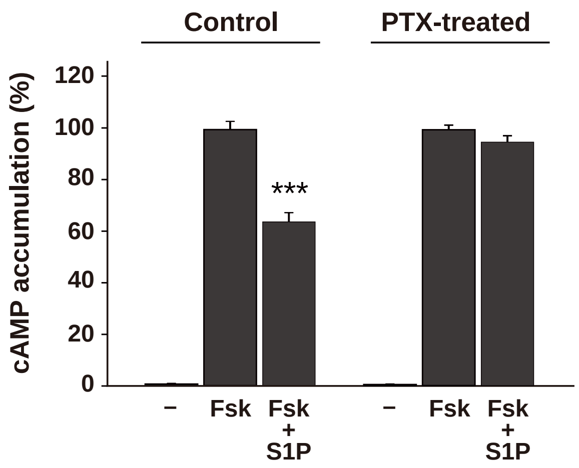

Supplementary Figure 9 PTX blocks sphingosine-1-phosphate (S1P)-induced inhibition of forskolin (Fsk)-stimulated cAMP accumulation in Flp-In TREx293 cells: a positive control for **Fig 3g.** Flp-In TREx293-Gpr176(tet-on)/Vipr2 cells were cultured with either PTX (100 ng/ml) or control vehicle for 16 h and stimulated with 50 μM Fsk in the presence or absence of 100 μM S1P for 15 min. cAMP levels were determined by enzyme immunoassays, and values were normalized to those of the cells treated with Fsk alone (100%). Data are the mean ± s.e.m. of four independent experiments. ***$P < 0.001$ vs. Fsk-stimulated control cells.

Supplementary Figure 10

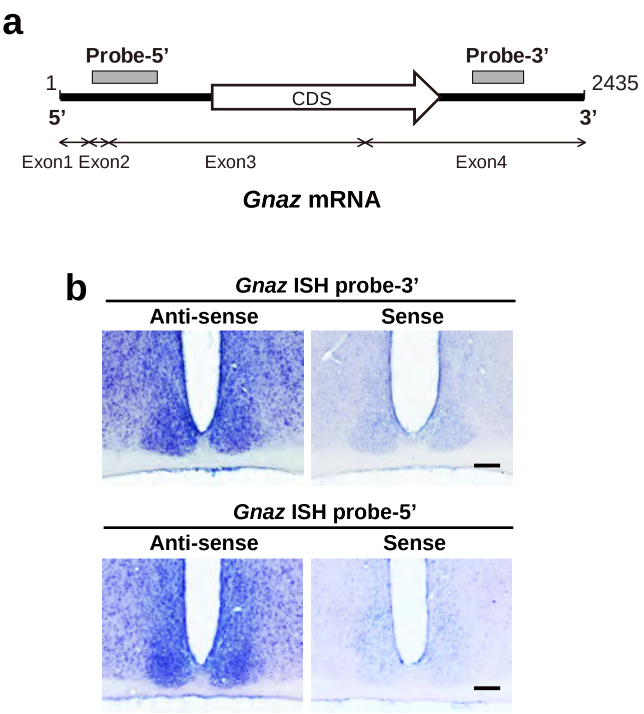

Supplementary Figure 10 Gz exists in the SCN. (**a**) A schematic structure of the mouse *Gnaz* transcript. Shaded boxes indicate the positions of gene-specific *in situ* hybridization probes that we designed in the 5'- and 3'-untranslated regions of this gene. Importantly, these regions diverge in sequence between Gz and the other Gi/o family member genes. CDS, coding sequence. (**b**) *In situ* hybridization of *Gnaz.* Shown are representative mouse coronal SCN sections *in situ* hybridized with digoxigenin-labeled 5'- and 3'-probes for *Gnaz*. Sense probes were used as negative controls. The photomicrographs for the 5'-probe are related to **Fig. 4a**. Scale bar, 100 μm.

Supplementary Figure 11

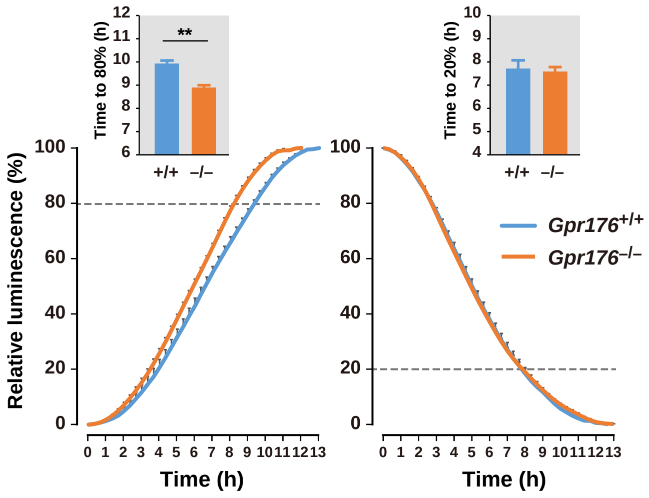

Supplementary Figure 11 Waveform alignments of rising and decreasing phase of *Per1-luc* activity in *Gpr176*$^{+/+}$ and *Gpr176*$^{-/-}$ organotypic SCN slices, examined in **Fig. 1j,k**. Left, traces aligned to trough time and normalized (trough = 0%, peak = 100%). Right, traces aligned to peak time and normalized. Normalized data of the second and third cycles of *Per1-luc* activity were averaged within a single slice and plotted as means ± s.e.m. from four separate SCN slices per genotype. Inserts show time to ascend to 80% (left) or decline to 20% (right) (means ± s.e.m., $n = 4$). $^{**}P < 0.01$, t-test. Note that the waveform analysis revealed an “asymmetric” period shortening by the deletion of *Gpr176*: *Gpr176* deficiency significantly accelerates the rising phase whereas over the decreasing phase it has little effect. It appears that the deletion of Gpr176 signaling allows faster rising of clock gene expression in the SCN.

Supplementary Figure 12

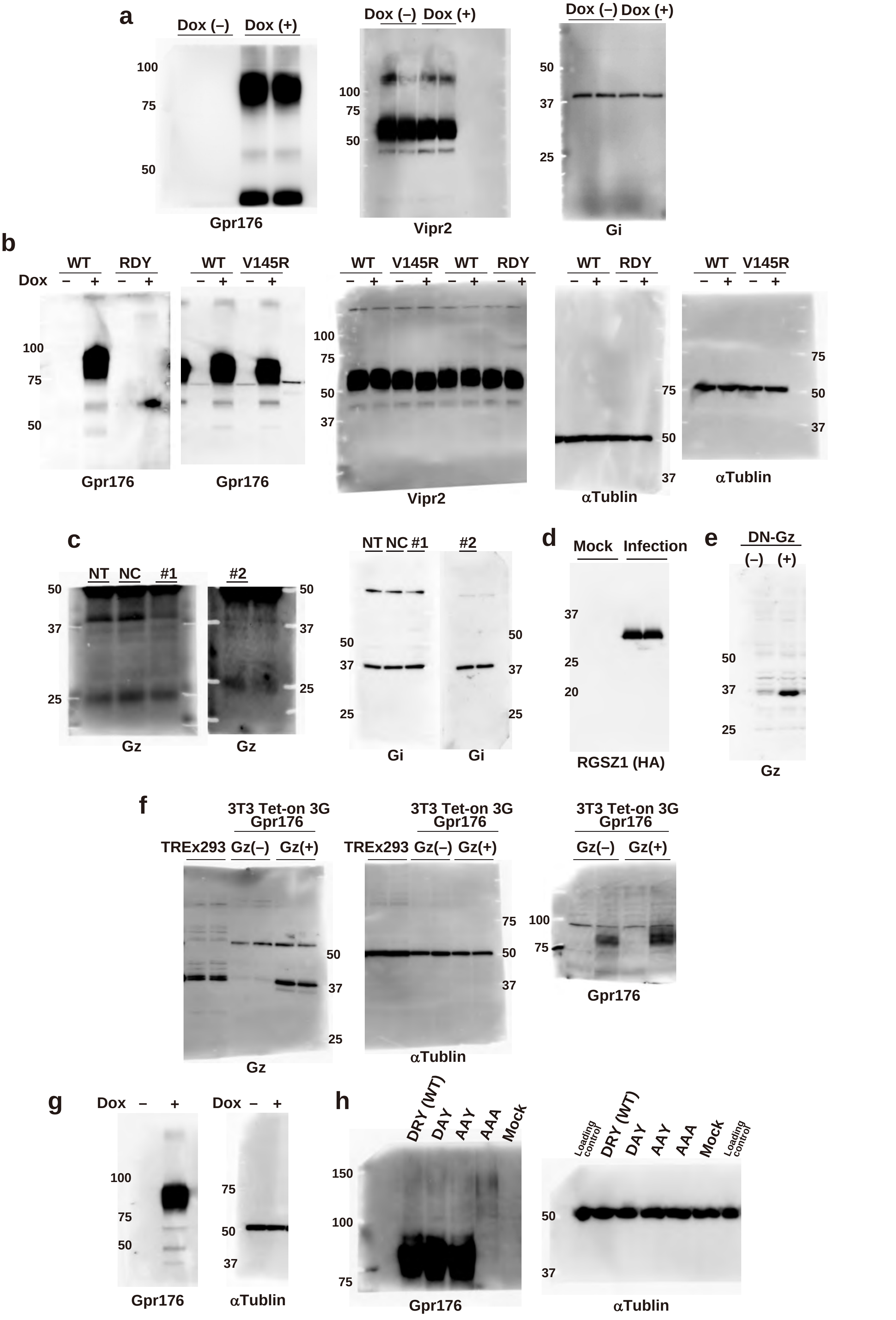

Supplementary Figure 12 Full version of Western blots shown in Fig. 3b (**a**), Fig. 3f (**b**), Fig. 4c (**c**), Fig. 4d (**d**), Fig. 4e (**e**), Fig. 4f (**f**), Supplementary Fig. 7a (**g**), and Supplementary Fig. 8c (**h**).
